# Cerebral perfusion pressure trajectories and cumulative exposure metrics predict in-hospital mortality in acute brain injury

**DOI:** 10.3389/fmed.2026.1838528

**Published:** 2026-05-22

**Authors:** Juan Wang, Hai-Bo Li, Man-Man Xu, Wen-Juan Li, Chun-Hua Hang, Peng-Lai Zhao

**Affiliations:** 1 Department of Neurosurgery, Nanjing Drum Tower Hospital, The Affiliated Nanjing Drum Tower Hospital of Nanjing University Medical School, Nanjing, China; 2 Department of Neurosurgery, Nanjing Drum Tower Hospital Clinical College of Nanjing University of Chinese Medicine, Nanjing, China; 3 Neurosurgical Institute, Nanjing University, Nanjing, China; 4 Fujian Maternity and Child Health Hospital, College of Clinical Medicine for Obstetrics and Gynecology and Pediatrics, Fujian Medical University, Fuzhou, China; 5 Center for Medical Big Data, Nanjing Drum Tower Hospital, The Affiliated Nanjing Drum Tower Hospital of Nanjing University Medical School, Nanjing, China

**Keywords:** acute brain injury, cerebral perfusion pressure, cumulative CPP metrics, in-hospital mortality, trajectory phenotype

## Abstract

**Objective:**

Fixed cerebral perfusion pressure (CPP) targets may not fully capture dynamic changes in acute brain injury (ABI). We aimed to identify CPP trajectory phenotypes, evaluate cumulative CPP metrics, and examine their associations with in-hospital mortality in ICU patients with ABI.

**Methods:**

This multicenter retrospective cohort study included 1,466 adults with ABI from three ICU databases (MIMIC-IV, eICU, and NSICU). CPP trajectory phenotypes were identified using latent class growth modeling. Cumulative CPP metrics were derived using threshold-specific methods at 50, 60, and 70 mmHg and a mixed-effects approach. Associations with in-hospital mortality were assessed using multivariable Cox regression and survival analyses. The incremental prognostic value of CPP trajectory was evaluated as a secondary exploratory analysis.

**Results:**

Four CPP trajectory phenotypes were identified: Stable Normal, Gradual Recovery, Labile Improvement, and Rapid Decline. Compared with Stable Normal, mortality risk increased progressively in Gradual Recovery (HR 1.720, 95% CI 1.252–2.362), Labile Improvement (HR 2.081, 95% CI 1.508–2.873), and Rapid Decline (HR 5.313, 95% CI 3.547–7.958; all *P* < 0.001). Higher cumulative CPP metrics derived from threshold-specific and mixed-effects approaches were consistently associated with lower in-hospital mortality (all *P* < 0.001). Survival analyses showed clear separation across phenotypes, with Stable Normal showing the highest survival probability and Rapid Decline the lowest. Adding CPP trajectory to a baseline model modestly improved discrimination and reclassification (AUC 0.759 to 0.773, *P* = 0.011; IDI 0.025; continuous NRI 0.157; both *P* < 0.001).

**Conclusions:**

Distinct CPP trajectory phenotypes and cumulative CPP metrics were associated with in-hospital mortality in ICU patients with ABI. CPP trajectory also provided modest incremental prognostic information and warrants prospective validation.

## Graphical Abstract

**Figure:**
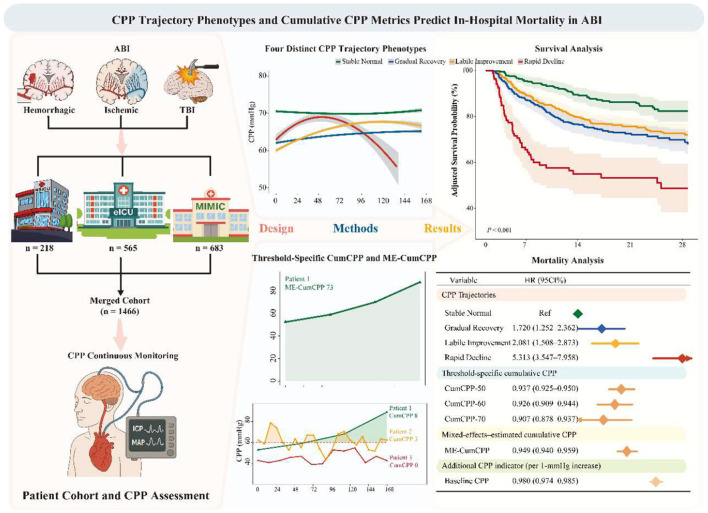
Schematic overview of cohort assembly, CPP trajectory phenotypes, survival analysis, and complementary CPP metrics in the merged ABI cohort. The forest plot displays associations of trajectory phenotypes, threshold-specific cumulative CPP, mixed–effects–estimated cumulative CPP, and baseline CPP with in–hospital mortality. Abbreviations: ABI, acute brain injury; CPP, cerebral perfusion pressure; CumCPP, cumulative CPP; ME–CumCPP, mixed–effects–estimated cumulative CPP.

## 1 Introduction

Acute brain injury (ABI), including traumatic brain injury (TBI), acute ischemic stroke (AIS), intracerebral hemorrhage (ICH), and subarachnoid hemorrhage (SAH), remains a major cause of death and long-term disability worldwide (1, 2). Stroke alone contributes to over 160 million disability-adjusted life years globally (3). In contrast, TBI predominantly affects younger populations and frequently leads to persistent neurocognitive and functional impairments (4). Patients with ABI frequently require intensive care management, wherein timely and effective prevention of secondary ischemic injury is essential for improving clinical outcomes (5).

Cerebral perfusion pressure (CPP), calculated as the difference between mean arterial pressure (MAP) and intracranial pressure (ICP), is a critical therapeutic target for maintaining adequate cerebral blood flow and preventing secondary injury (6, 7). Current clinical guidelines recommend maintaining CPP within a fixed target range to minimize ischemic risks (8, 9). However, accumulating evidence suggests substantial inter-individual variability in cerebrovascular autoregulation among patients with ABI (10). Consequently, fixed CPP thresholds may not adequately reflect individual dynamic physiological requirements (11). Additionally, variations in ICP monitoring practices and the absence of consensus on individualized ICP thresholds complicate personalized CPP management (12). Collectively, these observations highlight the limitations of static CPP targets and support further evaluation of dynamic CPP patterns in ABI.

Emerging analytical methods, particularly trajectory-based modeling using latent class growth modeling (LCGM), provide useful tools for characterizing longitudinal physiological patterns in critical care (13–15). In parallel, cumulative exposure metrics, such as area under the curve (AUC), integrate the magnitude and duration of physiological deviation over time and have shown prognostic value in ABI and related neurocritical conditions (16, 17). These approaches provide complementary information, with trajectory modeling capturing pattern heterogeneity and cumulative metrics summarizing longitudinal CPP exposure. However, few multicenter studies have jointly evaluated CPP trajectory phenotypes and cumulative CPP metrics within a broad ABI population.

In this multicenter retrospective cohort of ICU-admitted patients with ABI, we examined dynamic CPP phenotypes using trajectory modeling and cumulative CPP metrics. We evaluated their associations with in-hospital mortality and the incremental prognostic value of CPP trajectory beyond conventional clinical variables. This study extends prior dynamic CPP research to a broader ABI cohort and provides a basis for prognostic enrichment and future validation.

## 2 Methods

### 2.1 Data source

Data were obtained from: (1) the Neurosurgical ICU (NSICU) database of Nanjing Drum Tower Hospital, Clinical College of Nanjing Medical University (2024–2025); (2) the eICU Collaborative Research Database (version 2.0; 2014–2015) (18), which includes data from 335 hospitals across the United States; and (3) the Medical Information Mart for Intensive Care IV (MIMIC-IV, version 3.1; Beth Israel Deaconess Medical Center, 2008–2022) (19), accessed under certification ID 62674474.

### 2.2 Study population

This retrospective cohort study included adult patients with ABI due to TBI, AIS, ICH, or SAH, while excluding ABI cases due to tumors, infections, toxic encephalopathy, or other etiologies. Patients were eligible for inclusion if measurements of ICP and MAP were available. Only data from each patient's initial ICU admission during their first hospitalization were analyzed. In-hospital mortality was defined as the primary outcome. Patients were excluded if they met any of the following criteria: (1) ICU stay < 1 day; (2) age < 18 years; (3) missing outcome data; (4) missing ICP measurements; or (5) fewer than three valid paired ICP-MAP measurement pairs (Supplementary Figure 1).

### 2.3 Data extraction

Clinical data from the MIMIC-IV and eICU databases were extracted using structured SQL queries, while NSICU data were collected using electronic case report forms. Variables across all datasets were harmonized and categorized into demographic characteristics, physiological parameters, laboratory results, comorbidities, therapeutic interventions, discharge location, and hospital outcomes. Detailed definitions and coding schemes for all variables are available in Supplementary Table 1. The extent of missingness among baseline covariates is summarized in Supplementary Table 2. Missing baseline covariates were handled using multiple imputation by chained equations (MICE) with 10 imputed datasets. Diagnostic plots comparing observed and imputed values, observed and completed-data distributions, and trace plots assessing imputation stability across the 10 imputations are shown in Supplementary Figures 2–4.

### 2.4 Latent class growth modeling of CPP trajectories

CPP trajectory phenotypes were identified using LCGM *via* the hlme function in the R lcmm package. Hourly CPP values were derived from paired hourly mean arterial pressure and intracranial pressure recordings during the first 168 h after ICU admission. When more than one MAP or ICP value was available within the same hour, the hourly mean was used. To harmonize irregular sampling densities across the three databases and reduce short-term noise in retrospectively extracted bedside measurements, hourly CPP data were averaged into consecutive 5-h bins. The distribution of valid CPP observations per patient and the temporal availability of CPP data across the first 168 h are shown in Supplementary Figure 5. Implausible values (< 0 or >150 mmHg) were excluded, and bins lacking valid paired data were treated as missing. No imputation was applied for intermittent missing CPP values at other time points, and trajectory estimation was based on all available observed CPP bins for each patient. Trajectories were modeled using a quadratic time function with class-specific fixed effects for time and subject-specific random intercepts. Models with two to six latent classes were fitted and compared. The optimal model was selected based on an integrated assessment of statistical fit, including the Akaike information criterion (AIC), Bayesian information criterion (BIC), and sample-size adjusted BIC (SABIC), as well as entropy, mean posterior probabilities, class proportions, and clinical interpretability, with solutions containing very small classes (< 5% of the cohort) considered unstable and not preferred (14, 15). Sensitivity analyses were additionally performed using alternative observation windows (72 and 120 h) and temporal aggregation schemes (4-h and 8-h bins).

### 2.5 Threshold-specific cumulative CPP calculation

Threshold-specific cumulative CPP metrics were calculated to quantify the cumulative amount of CPP maintained above predefined thresholds during the first 168 h after ICU admission. Thresholds of 50, 60, and 70 mmHg were selected according to established clinical recommendations and commonly used CPP targets. Based on the 5-h binned CPP series, cumulative CPP at each threshold was calculated using trapezoidal integration after truncating CPP values below the corresponding threshold to that threshold, such that only values above the threshold contributed to the cumulative measure (20). For each threshold, both total threshold-specific cumulative CPP (T-CumCPP) and mean threshold-specific cumulative CPP (M-CumCPP) were derived. Because T-CumCPP is inherently influenced by the number of available observations and the effective observation window, M-CumCPP was calculated by dividing T-CumCPP by the number of valid 5-h intervals contributing to that metric, thereby reducing dependence on unequal observation windows across patients. Only M-CumCPP was retained in the main association analyses. Illustrative examples at thresholds of 50 and 60 mmHg are shown in Supplementary Figures 6A and 6B.

### 2.6 Mixed-effects–estimated cumulative CPP calculation

Mixed-effects–estimated cumulative CPP was derived from repeated 5-h binned CPP measurements obtained during the first 168 h after ICU admission. A linear mixed-effects model was fitted with time as a fixed effect and subject-specific random effects to account for within-patient correlation and between-patient variability in repeated CPP measurements. Patient-specific smoothed CPP trajectories were then generated from the fitted model, and cumulative CPP was quantified by trapezoidal integration of each smoothed trajectory over the available observation window (17, 21). Both total mixed-effects–estimated cumulative CPP (T-ME-CumCPP) and mean mixed-effects–estimated cumulative CPP (M-ME-CumCPP) were derived. To reduce the influence of unequal observation windows related to early death or discharge, M-ME-CumCPP was calculated by dividing T-ME-CumCPP by the number of valid 5-h intervals contributing to the estimate. Only M-ME-CumCPP was retained in the main results. Illustrative examples are shown in Supplementary Figures 6C–E.

### 2.7 Exploratory assessment of the incremental prognostic value of CPP trajectory

As a secondary exploratory prognostic analysis, we examined whether CPP trajectory was consistently retained across multiple feature-selection approaches and whether it provided incremental prognostic information beyond conventional clinical variables. Candidate predictors were evaluated using three complementary methods: the Boruta algorithm, stepwise regression, and best subset selection (BSS). Boruta provided variable-importance ranking, whereas BSS evaluated alternative variable combinations using Mallows' Cp, BIC, adjusted *R*^2^, and *R*^2^. Predictors selected repeatedly across methods were used to define a baseline prognostic model without CPP trajectory. A trajectory-enhanced model was then constructed by adding CPP trajectory to the baseline model.

Incremental prognostic value was assessed primarily using AUC, integrated discrimination improvement (IDI), continuous net reclassification improvement (NRI), and median improvement in predicted risk scores. Internal validation and calibration were evaluated as supplementary analyses.

### 2.8 Statistical analysis

Continuous variables were assessed for normality using the Kolmogorov–Smirnov test and presented as mean ± SD or median (IQR) for normally or non-normally distributed data, respectively. Categorical variables were reported as frequencies (%). Continuous variables were compared between groups using independent *t*-tests or Mann–Whitney U tests, as appropriate, while categorical variables were compared using Chi-square or Fisher's exact tests. For comparisons across more than two groups, continuous variables were analyzed using one-way analysis of variance (ANOVA) or the Kruskal–Wallis test, followed by appropriate *post hoc* comparisons when indicated.

Cox proportional hazards models were used to evaluate the associations of CPP trajectory phenotypes and cumulative CPP metrics with in-hospital mortality. Three models were constructed: Model 1 was unadjusted; Model 2 was partially adjusted for a parsimonious set of prespecified covariates selected according to clinical relevance and cross-database availability, with change-in-estimate (CIE) screening and collinearity diagnostics used as supplementary assessments. Model 3 was fully adjusted by further incorporating additional prespecified comorbidities, neurologic severity indicators, and treatment variables. The proportional hazards assumption was assessed using Schoenfeld residuals.

Unadjusted Kaplan–Meier curves, covariate-adjusted survival curves estimated from the fully adjusted Cox model, and landmark survival curves were generated to visualize survival differences across CPP trajectory phenotypes. Survival curves were displayed over the first 28 days after ICU admission to focus on the early in-hospital event pattern, where most events occurred. Because CPP trajectory phenotypes were derived from measurements collected during the first 168 h after ICU admission, a 3-day landmark analysis together with Cox analyses using 3-day and 5-day time stratification was additionally performed to examine the temporal robustness of the observed associations. Subgroup analyses were performed across clinically relevant strata, including source, TBI, ventilation, vasopressor use, hemorrhage status, age (< 65 vs. ≥65 years), sex, craniotomy, hypertension, and diabetes. Sensitivity analyses additionally included E-value estimation to assess the robustness of the main associations to potential unmeasured confounding. Statistical analyses were performed using *R* (version 4.2.2) and the Free Statistics analysis platform (22). Statistical significance was set at *P* < 0.05.

## 3 Results

A total of 22,207 patients diagnosed with ABI were initially identified from the three ICU databases (MIMIC-IV version 3.1, eICU version 2.0, and the NSICU dataset). After applying predefined eligibility criteria, 1,466 patients were included in the final analytic cohort (Supplementary Figure 1).

### 3.1 CPP trajectory phenotypes in ICU-admitted ABI patients

A four-class latent trajectory model was retained as the primary solution based on the overall balance among statistical fit, class distribution, posterior classification probabilities, and clinical interpretability (Supplementary Table 3). Although models with more classes yielded lower information criteria, they generated very small classes and less clinically stable subclass structures.

**Figure 1:**
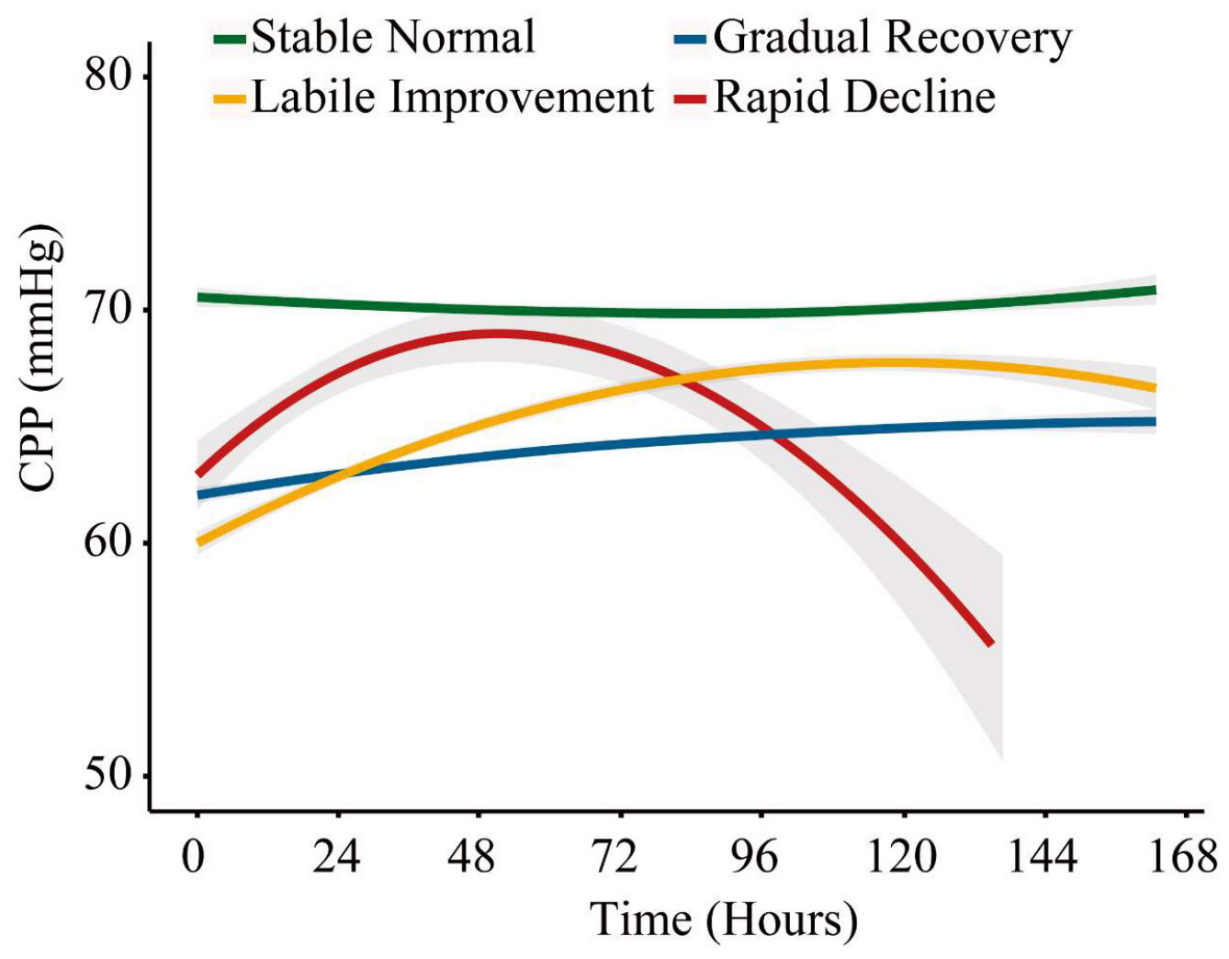
Four distinct CPP trajectory phenotypes. *Legend:* Modeled CPP trajectories with 95% confidence intervals (shaded regions) over the first 168 h after ICU admission. Trajectory 1, Stable Normal (green), remained relatively stable around 70 mmHg. Trajectory 2, Gradual Recovery (blue), increased gradually from a lower initial level. Trajectory 3, Labile Improvement (yellow), started lower, rose more rapidly, and then leveled off. Trajectory 4, Rapid Decline (red), showed an early rise followed by a marked decline.

The four CPP trajectory phenotypes were characterized as follows (Figure 1): Trajectory 1, Stable Normal (SN), remained relatively stable around 70 mmHg throughout the observation period. Trajectory 2, Gradual Recovery (GR), started from a lower CPP level and increased gradually over time. Trajectory 3, Labile Improvement (LI), began at a lower level, rose more rapidly during the early phase, and then stabilized with a slight decline toward the end of follow-up. Trajectory 4, Rapid Decline (RD), showed an early rise followed by a marked decline, with persistently lower CPP levels during later time periods. Sensitivity analyses using alternative observation windows showed that the overall trajectory patterns remained broadly consistent (Supplementary Figure 7).

### 3.2 Baseline characteristics stratified by CPP trajectories

Baseline characteristics were compared across the four CPP trajectory groups to further define their clinical profiles (Table 1). Differences were observed in demographic features, ABI subtype composition, physiologic measures, illness severity, major interventions, and in-hospital mortality across the four groups. Overall, 359 of 1,466 patients (24.5%) died in hospital, including 57/421 (13.5%) in SN, 133/514 (25.9%) in GR, 118/439 (26.9%) in LI, and 51/92 (55.4%) in RD (Table 1). In particular, age, TBI prevalence, baseline CPP, initial ICP, SOFA score, APACHE III score, vasopressor use, and mechanical ventilation varied significantly across groups (all *P* < 0.05). The distribution of trajectory phenotypes also differed by ABI subtype and data source (Supplementary Figure 8).

**Table 1:** Baseline characteristics stratified by CPP trajectory phenotypes.

Variables	Total (*n* = 1,466)	Stable normal (*n* = 421)	Gradual recovery (*n* = 514)	Labile improvement (*n* = 439)	Rapid decline (*n* = 92)	*P*
Age, years	56.0 ± 17.1	57.5 ± 14.6	56.4 ± 18.4	54.8 ± 17.9	52.3 ± 15.5	0.018
Sex, male, *n* (%)	801 (54.6)	221 (52.5)	305 (59.3)	230 (52.4)	45 (48.9)	0.058
BMI, kg/m^2^	27.8 ± 7.0	27.7 ± 7.5	28.3 ± 7.4	27.5 ± 6.2	27.3 ± 6.6	0.248
TBI, *n* (%)	420 (28.6)	79 (18.8)	178 (34.6)	122 (27.8)	41 (44.6)	< 0.001
Admission type, *n* (%)	1,178 (80.4)	365 (86.7)	403 (78.4)	347 (79.0)	63 (68.5)	< 0.001
Admission time, *n* (%)	492 (33.6)	151 (35.9)	170 (33.1)	138 (31.4)	33 (35.9)	0.538
Respiratory rate, bpm	20.5 ± 9.3	19.2 ± 6.8	20.6 ± 9.3	20.9 ± 10.7	23.5 ± 10.9	< 0.001
Heart rate, bpm	91.7 ± 25.3	89.4 ± 20.9	90.6 ± 25.0	92.7 ± 27.0	103.0 ± 33.5	< 0.001
MAP, mmHg	91.9 ± 30.3	97.1 ± 23.8	90.0 ± 30.2	86.9 ± 32.8	101.7 ± 39.0	< 0.001
Temperature,°C	36.8 ± 0.9	36.8 ± 0.7	36.8 ± 0.9	36.8 ± 1.0	36.4 ± 1.1	0.001
Urine output, mL	1,981.4 (1,365.0, 2934.1)	1,822.8 (1,278.4, 2,597.5)	1,959.7 (1,366.2, 2,931.7)	2,092.8 (1,370.9, 3,079.5)	2,938.7 (1,717.6, 4,556.4)	< 0.001
ALT, U/L	24.0 (15.3, 39.0)	21.2 (14.1, 34.9)	23.8 (16.0, 39.0)	25.0 (16.0, 46.0)	27.0 (18.8, 43.5)	< 0.001
BUN, mg/dL	17.1 ± 9.0	16.9 ± 8.5	17.5 ± 9.5	17.1 ± 8.9	16.3 ± 7.7	0.535
Creatinine, mg/dL	0.9 (0.7, 1.1)	0.8 (0.7, 1.1)	0.9 (0.7, 1.2)	0.9 (0.7, 1.1)	0.9 (0.7, 1.2)	0.007
Glucose, mg/dL	154.0 ± 73.5	143.9 ± 46.3	160.5 ± 100.9	153.4 ± 56.0	166.4 ± 61.1	0.002
Sodium, mmol/L	143.0 ± 5.6	142.2 ± 4.6	143.2 ± 5.7	142.8 ± 5.0	146.2 ± 9.6	< 0.001
Potassium, mmol/L	3.6 ± 0.5	3.6 ± 0.4	3.6 ± 0.5	3.6 ± 0.5	3.5 ± 0.5	0.031
RBC, × 10^12^/L	3.8 ± 0.8	3.9 ± 0.7	3.7 ± 0.8	3.8 ± 0.8	3.6 ± 0.8	0.005
WBC, × 10^9^/L	15.3 ± 6.6	14.2 ± 7.1	15.2 ± 5.7	15.9 ± 6.7	17.7 ± 8.2	< 0.001
Platelet, × 10^9^/L	195.0 ± 77.0	199.5 ± 75.0	190.6 ± 76.7	194.9 ± 76.9	198.6 ± 87.2	0.341
INR	1.2 ± 0.6	1.2 ± 0.8	1.2 ± 0.5	1.2 ± 0.5	1.3 ± 0.5	0.294
Hypertension, *n* (%)	766 (52.3)	260 (61.8)	257 (50.0)	210 (47.8)	39 (42.4)	< 0.001
Diabetes, *n* (%)	198 (13.5)	49 (11.6)	89 (17.3)	50 (11.4)	10 (10.9)	0.020
Stroke History, *n* (%)	134 (9.1)	57 (13.5)	41 (8.0)	27 (6.2)	9 (9.8)	0.001
Liver Disease, *n* (%)	71 (4.8)	29 (6.9)	17 (3.3)	22 (5.0)	3 (3.3)	0.084
CCI	3.0 (1.0, 5.0)	3.0 (2.0, 5.0)	3.0 (1.0, 5.0)	2.0 (1.0, 4.0)	1.5 (0.0, 3.2)	< 0.001
Initial GCS	7.5 ± 4.2	7.0 ± 4.1	7.7 ± 4.2	7.7 ± 4.1	7.2 ± 4.6	0.025
Initial ICP, mmHg	9.0 (5.0, 14.0)	7.0 (2.0, 11.0)	9.0 (5.0, 14.0)	11.0 (6.0, 16.0)	10.0 (5.0, 31.0)	< 0.001
Baseline CPP, mmHg	60.0 ± 17.0	74.1 ± 11.7	59.0 ± 10.1	51.1 ± 15.4	44.2 ± 27.4	< 0.001
SOFA score	4.4 ± 2.8	3.8 ± 2.3	4.5 ± 2.9	4.8 ± 2.9	5.2 ± 3.2	< 0.001
APACHE III score	50.1 ± 24.9	43.6 ± 22.1	50.9 ± 24.6	52.2 ± 25.2	64.6 ± 28.7	< 0.001
Dialysis, *n* (%)	26 (1.8)	6 (1.4)	11 (2.1)	5 (1.1)	4 (4.3)	0.165
Vasopressor, *n* (%)	362 (24.7)	67 (15.9)	123 (23.9)	132 (30.1)	40 (43.5)	< 0.001
Mannitol, *n* (%)	321 (21.9)	95 (22.6)	119 (23.2)	84 (19.1)	23 (25.0)	0.382
Ventilation, *n* (%)	975 (66.5)	249 (59.1)	350 (68.1)	309 (70.4)	67 (72.8)	0.001
Craniotomy, *n* (%)	932 (63.6)	289 (68.6)	322 (62.6)	274 (62.4)	47 (51.1)	0.010
Embolization, *n* (%)	409 (27.9)	110 (26.1)	124 (24.1)	155 (35.3)	20 (21.7)	< 0.001
In-hospital mortality, *n* (%)						< 0.001
No	1,107 (75.5)	364 (86.5)	381 (74.1)	321 (73.1)	41 (44.6)	
Yes	359 (24.5)	57 (13.5)	133 (25.9)	118 (26.9)	51 (55.4)	

Data are presented as mean ± SD, median (IQR), or n (%), as appropriate. Urine output, vasopressor use, ventilation, and mannitol use were recorded within the first 24 h after ICU admission.

SN included relatively older patients and was characterized by the highest prevalence of hypertension and prior stroke, the lowest illness severity, and the highest baseline CPP. GR showed intermediate clinical severity and a relatively high prevalence of TBI. LI comprised younger patients and was characterized by higher initial ICP, lower baseline CPP, and greater use of embolization. RD exhibited the highest overall clinical severity, with the greatest prevalence of TBI, the highest SOFA and APACHE III scores, the lowest baseline CPP, and the highest rates of vasopressor use and mechanical ventilation. Comparisons of ICP, CPP, GCS, and discharge location across trajectory phenotypes are shown in Supplementary Figure 9.

### 3.3 Associations between CPP trajectories, CPP metrics, and in-hospital mortality

We evaluated the associations of CPP trajectory phenotypes and complementary CPP metrics with in-hospital mortality using progressively adjusted Cox models (Table 2). Exploratory covariate screening results are summarized in Supplementary Table 4; however, the final adjustment sets were determined primarily by prespecified clinical relevance and cross-database availability. Model 2 adjusted for age, sex, TBI, temperature, urine output, ALT, BUN, glucose, and sodium, whereas Model 3 further adjusted for hypertension, diabetes, initial GCS, mannitol use, vasopressor use, ventilation, and craniotomy. In the fully adjusted model (Model 3), compared with SN, mortality risk increased progressively across the other trajectory phenotypes, with HRs of 1.720 (95% CI 1.252–2.362; *P* < 0.001) for GR, 2.081 (95% CI 1.508–2.873; *P* < 0.001) for LI, and 5.313 (95% CI 3.547–7.958; *P* < 0.001) for RD. A similar gradient was observed in the partially adjusted and unadjusted models.

**Table 2:** Associations of CPP trajectory phenotypes and complementary CPP metrics with in-hospital mortality.

Categories	Model 1		Model 2		Model 3	
	HR (95% CI)	*P*	HR (95% CI)	*P*	HR (95% CI)	*P*
CPP trajectories						
Stable normal	Ref	—	Ref	—	Ref	—
Gradual recovery	1.981 (1.452–2.703)	< 0.001	1.737 (1.267–2.381)	< 0.001	1.720 (1.252–2.362)	< 0.001
Labile improvement	2.128 (1.551–2.920)	< 0.001	2.160 (1.569–2.972)	< 0.001	2.081 (1.508–2.873)	< 0.001
Rapid decline	6.114 (4.188–8.926)	< 0.001	5.427 (3.631–8.110)	< 0.001	5.313 (3.547–7.958)	< 0.001
Threshold-specific cumulative CPP						
CumCPP-50	0.936 (0.924–0.948)	< 0.001	0.938 (0.926–0.951)	< 0.001	0.937 (0.925–0.950)	< 0.001
CumCPP-60	0.923 (0.906–0.940)	< 0.001	0.927 (0.910–0.944)	< 0.001	0.926 (0.909–0.944)	< 0.001
CumCPP-70	0.896 (0.869–0.925)	< 0.001	0.908 (0.880–0.938)	< 0.001	0.907 (0.878–0.937)	< 0.001
Mixed-effects–estimated cumulative CPP						
ME-CumCPP	0.945 (0.937–0.954)	< 0.001	0.948 (0.939–0.958)	< 0.001	0.949 (0.940–0.959)	< 0.001
Additional CPP indicator (per 1-mmHg increase)						
Baseline CPP	0.977 (0.971–0.982)	< 0.001	0.978 (0.972–0.984)	< 0.001	0.980 (0.974–0.985)	< 0.001

Cox proportional hazards models were used to examine associations with in-hospital mortality, with Stable Normal phenotype as the reference. Model 1 was unadjusted; Model 2 adjusted for age, sex, TBI, temperature, urine output, ALT, BUN, glucose, and sodium; Model 3 further adjusted for hypertension, diabetes, initial GCS, mannitol use, vasopressor use, ventilation, and craniotomy. Abbreviations: CumCPP-50/60/70, threshold-specific cumulative CPP calculated at thresholds of 50/60/70 mmHg; ME-CumCPP, mixed-effects–estimated cumulative CPP.

Higher cumulative CPP metrics were consistently associated with lower in-hospital mortality. In Model 3, CumCPP-50, CumCPP-60, and CumCPP-70 were each inversely associated with mortality, with HRs of 0.937 (95% CI 0.925–0.950), 0.926 (95% CI 0.909–0.944), and 0.907 (95% CI 0.878–0.937), respectively (all *P* < 0.001). Similarly, higher ME-CumCPP was associated with reduced mortality risk (HR 0.949, 95% CI 0.940–0.959; *P* < 0.001). Baseline CPP also remained inversely associated with in-hospital mortality in the fully adjusted model (HR 0.980, 95% CI 0.974–0.985; *P* < 0.001). The robustness of the main associations to potential unmeasured confounding is further illustrated by the *E*-values shown in Supplementary Figure 11.

**Figure 2:**
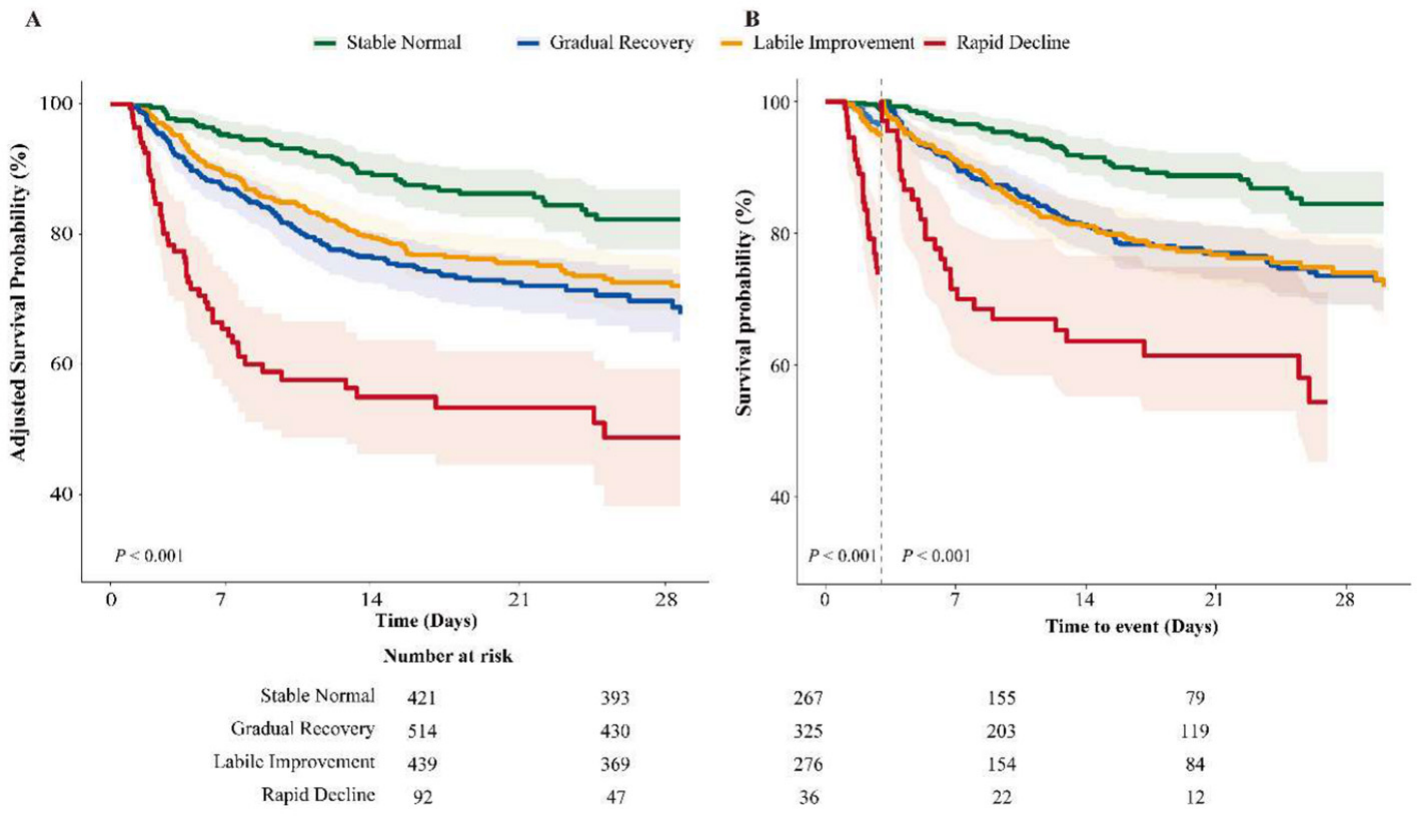
Survival curves and landmark analysis by CPP trajectory phenotypes. Panel **(A)** shows covariate-adjusted 28-day survival curves across the four CPP trajectory phenotypes. Panel **(B)** shows the 3-day landmark analysis restricted to patients surviving beyond day 3; the dashed line marks the landmark time point. Shaded areas indicate 95% confidence intervals. Stable Normal showed the highest survival probability, whereas Rapid Decline showed the lowest.

Kaplan–Meier analyses demonstrated clear survival separation across the four trajectory phenotypes. In the unadjusted curves (Supplementary Figure 10) and covariate-adjusted 28-day survival curves (Figure 2A), SN consistently showed the highest survival probability, whereas RD showed the lowest. GR and LI exhibited intermediate survival patterns, with LI generally showing lower survival than GR. In the 3-day landmark analysis restricted to patients surviving beyond day 3, the overall trajectory gradient remained evident (Figure 2B). Time-stratified Cox analyses showed generally consistent associations across clinically relevant follow-up intervals (Supplementary Table 5, Supplementary Figure 12).

### 3.4 Subgroup analyses of associations between CPP trajectories and in-hospital mortality

**Figure 3:**
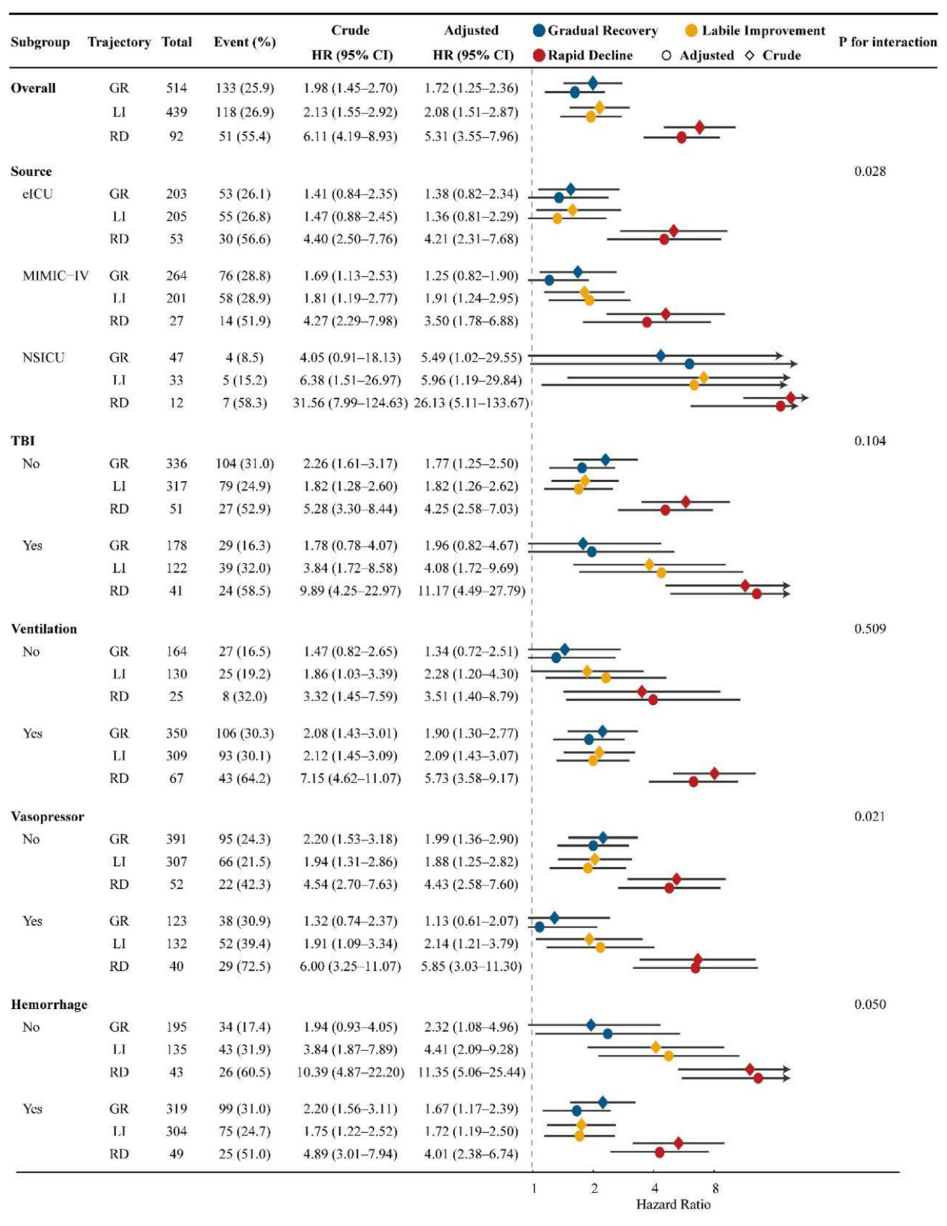
Subgroup Analysis by CPP Trajectory Phenotypes. *Legend:* Subgroup analyses of in-hospital mortality across CPP trajectory phenotypes, with Stable Normal as the reference. Subgroups were defined by source, TBI, ventilation, vasopressor use, and hemorrhage status. Diamonds indicate crude hazard ratios and circles indicate fully adjusted hazard ratios; horizontal lines represent 95% confidence intervals. Interaction *P* values are shown for each subgroup category.

Subgroup analyses showed broadly consistent associations between CPP trajectory phenotypes and in-hospital mortality. Across nearly all strata, RD consistently exhibited the highest mortality risk relative to SN, whereas GR and LI showed intermediate risk elevations. Although the effect sizes varied across subgroups and nominal interaction signals were observed in selected strata, the overall trajectory gradient remained preserved, with SN showing the lowest risk and RD the highest (Figure 3; Supplementary Figure 13).

### 3.5 Exploratory incremental prognostic value of CPP trajectory for in-hospital mortality

**Figure 4:**
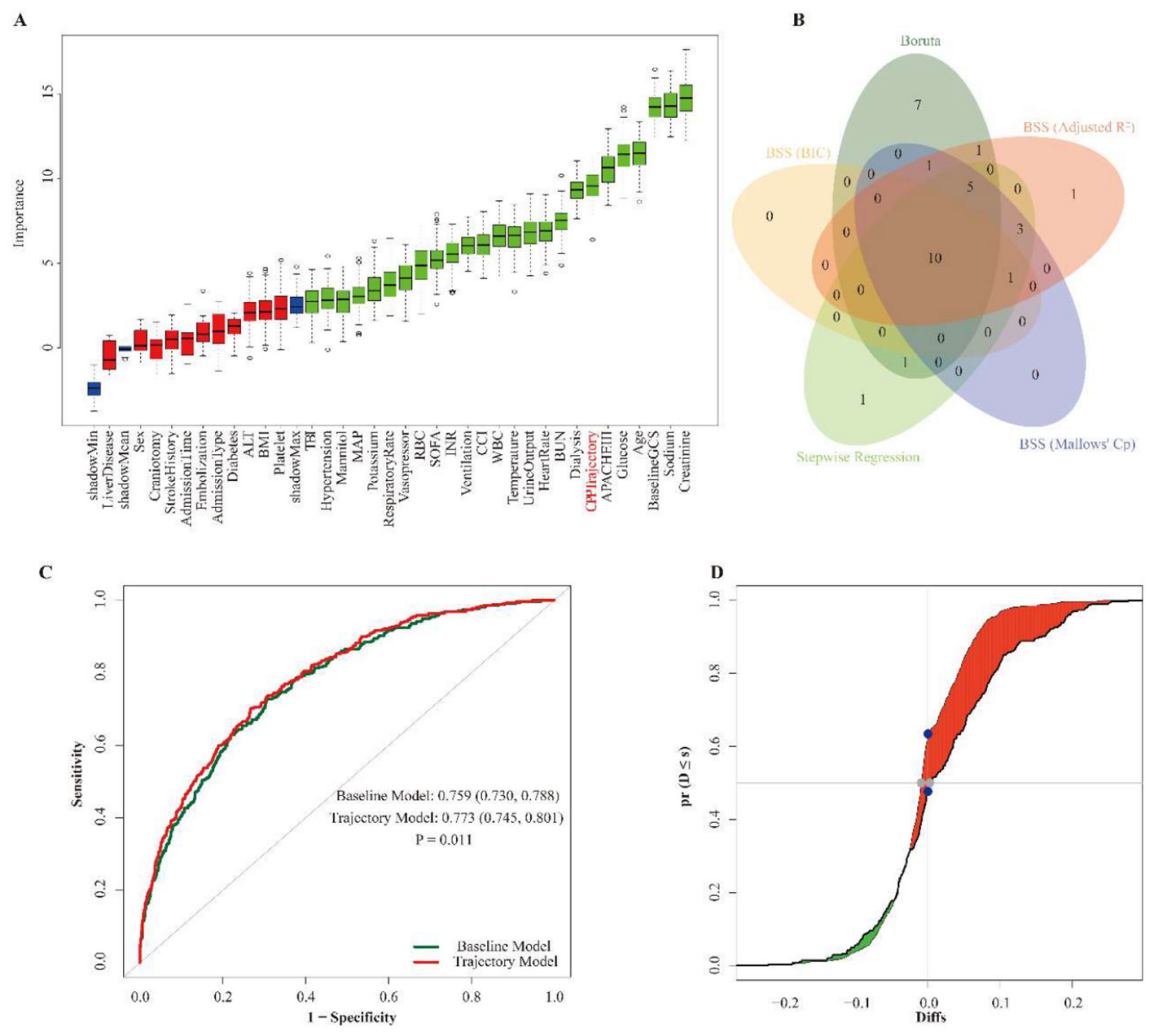
Feature Selection and Predictive Increment of CPP Trajectory. *Legend:* **(A)** Boruta variable importance plot. **(B)** Overlap of variables selected by Boruta, stepwise regression, and best subset selection. **(C)** ROC curves for the baseline and trajectory-enhanced models. **(D)** Reclassification after adding CPP trajectory, showing IDI and continuous NRI.

As a secondary exploratory prognostic analysis, we evaluated whether CPP trajectory provided incremental prognostic information beyond conventional clinical variables. Across multiple feature-selection approaches, CPP trajectory was consistently retained. In Boruta analysis, CPP trajectory showed high variable importance (Figure 4A). It was also retained in the overlap of variables selected by Boruta, stepwise regression, and best subset selection (Figure 4B), was included in the optimal subsets under all four BSS criteria (Supplementary Figure 14), and showed high consensus and frequent co-selection with other key predictors across methods (Supplementary Figure 15).

To assess incremental prognostic value, a trajectory-enhanced model was compared with a baseline model composed of conventional clinical predictors (Table 3). Adding CPP trajectory produced a modest but statistically significant improvement in model performance, increasing the AUC from 0.759 (95% CI 0.730–0.788) to 0.773 (95% CI 0.745–0.801; *P* = 0.011; Figure 4C, Table 3). Significant improvements were also observed for IDI (0.025, 95% CI 0.011–0.043; *P* < 0.001), continuous NRI (0.157, 95% CI 0.069–0.225; *P* < 0.001), and median improvement in predicted risk score (0.012, 95% CI 0.004–0.041; *P* < 0.001; Figure 4D, Table 3). Supplementary analyses further showed acceptable discrimination and calibration, with decision-curve and clinical impact analyses provided in Supplementary Figures 16 and 17. Overall, these findings suggest that CPP trajectory contributes incremental prognostic information, although the magnitude of improvement was modest.

**Table 3:** Incremental predictive value of CPP trajectory for in-hospital mortality.

Models	IDI		NRI (continuous)		Median improvement in risk score		AUC	
	Estimate (95% CI)	*P*	Estimate (95% CI)	*P*	Estimate (95% CI)	*P*	(95% CI)	*P*
Baseline model	Ref	—	Ref	—	Ref	—	0.759 (0.730–0.788)	—
Trajectory model	0.025 (0.011–0.043)	< 0.001	0.157 (0.069–0.225)	< 0.001	0.012 (0.004–0.041)	< 0.001	0.773 (0.745–0.801)	0.011

Predictive performance comparison between the Baseline model (Age, Urine Output, Sodium, WBC, Hypertension, APACHE III, Dialysis, Vasopressor, ventilation) and Trajectory-enhanced model (Baseline variables plus CPP Trajectory). The Baseline model was the reference to evaluate incremental improvements from CPP Trajectory inclusion.

## 4 Discussion

In this multicenter retrospective cohort of ICU-admitted patients with ABI, we identified four distinct CPP trajectory phenotypes with different clinical characteristics and in-hospital mortality risks. Relative to SN, GR, LI, and especially RD were associated with progressively higher mortality. Higher CumCPP, ME-CumCPP, and baseline CPP were each associated with lower in-hospital mortality. CPP trajectory was also repeatedly retained across feature-selection methods and provided modest incremental prognostic information beyond conventional clinical variables.

Continuous CPP monitoring remains an important component of neurocritical care because it provides information beyond isolated static measurements and reflects the evolving hemodynamic state of patients with ABI (7, 23). Current guidelines, such as those from the Brain Trauma Foundation, recommend maintaining CPP within a fixed target range, typically 60–70 mmHg, and adherence to these targets has been associated with favorable neurological outcomes (24, 25). However, accumulating evidence from stroke and TBI studies suggests that fixed CPP targets may not fully capture temporal fluctuations or between-patient heterogeneity in cerebrovascular physiology (26). In this context, dynamic CPP patterns may complement conventional threshold-based assessment by capturing longitudinal information beyond single time-point measurements. Prior studies using autoregulation-informed metrics, such as individualized optimal CPP, further support the potential value of dynamic monitoring approaches (23, 27). Our findings further support the relevance of dynamic CPP assessment in ABI and provide context for interpreting CPP trajectory phenotypes in relation to prognosis (26, 28).

Trajectory-based modeling provides a useful framework for characterizing longitudinal CPP patterns in addition to conventional static thresholds (14, 29, 30). Although this approach is not entirely new in neurocritical care, prior studies have shown that trajectory-based analyses can capture clinically relevant temporal heterogeneity in several neurologic conditions, including sepsis-associated encephalopathy, post-stroke cognitive impairment, and TBI recovery (31–33). Building on this prior work, our study extends trajectory-based CPP analysis to a broader and more heterogeneous cohort of ICU-admitted patients with ABI, and further evaluates these trajectory phenotypes together with cumulative CPP metrics.

The four CPP trajectory phenotypes identified in our study were associated with distinct clinical profiles and mortality risks. SN showed lower illness severity and the most favorable outcomes, whereas RD was characterized by greater clinical severity and the poorest prognosis; GR and LI exhibited intermediate risk profiles. These findings suggest that longitudinal CPP patterns capture clinically relevant heterogeneity in ABI and may summarize differences in overall severity, treatment intensity, and disease composition over time (8, 34). The preservation of the overall risk gradient across subgroup and time-stratified analyses further supports the prognostic relevance of these trajectory patterns.

Taken together, these findings suggest that trajectory-based CPP phenotyping may improve risk stratification by incorporating longitudinal information beyond single CPP measurements. However, the incremental prognostic value beyond conventional clinical variables was modest, and these results should therefore be interpreted as supportive rather than practice-changing. At present, the findings are best viewed as providing a basis for further prospective validation of dynamic CPP phenotyping within real-time multimodal monitoring frameworks.

Threshold-specific CumCPP and ME-CumCPP provide complementary ways to summarize longitudinal CPP information beyond single time-point measurements (20, 35). CumCPP reflects the amount and duration of CPP maintained above prespecified thresholds over time, whereas ME-CumCPP is derived from smoothed individual CPP trajectories and provides a smoothed summary of overall longitudinal CPP patterns (16, 21). Both approaches summarize cumulative longitudinal CPP information in neurocritical care and may offer additional prognostic information beyond isolated measurements (36, 37). Prior studies in TBI and cerebrovascular monitoring have likewise suggested that cumulative ICP or CPP metrics may be clinically informative (12, 28).

In our cohort, higher CumCPP and ME-CumCPP were consistently associated with lower in-hospital mortality, complementing the findings from trajectory-based analyses. These associations suggest that longitudinal CPP patterns, including the extent to which CPP is maintained above clinically relevant thresholds, carry prognostic information not captured by baseline CPP alone. Together, these findings support the potential value of cumulative CPP assessment for risk stratification and further prospective evaluation (8, 12).

This multicenter retrospective study has several limitations. First, the retrospective design precludes causal inference and leaves room for residual confounding, while the requirement for ICP monitoring may have introduced selection bias toward a more severely ill and intensively monitored population. Second, differences in data recording, monitoring practices, and clinical management across the three databases may have contributed to residual heterogeneity despite harmonization (18, 19). Third, because CPP trajectories and cumulative CPP metrics were constructed from measurements obtained during the first 168 h after ICU admission, bias related to unequal survival or observation windows cannot be fully excluded. In addition, subtype-specific differences in pathophysiology, monitoring context, and treatment intensity may have contributed to the observed associations, and the identified trajectory classes may partly reflect these differences rather than distinct biologic phenotypes alone. Finally, the analysis was limited to in-hospital mortality and did not capture long-term neurologic or functional recovery. Future prospective studies with more granular clinical characterization and longitudinal outcomes are needed to validate these findings.

## 5 Conclusion

In this multicenter retrospective study, distinct CPP trajectory phenotypes and cumulative CPP metrics were associated with in-hospital mortality in ICU-admitted patients with ABI. CPP trajectory also provided modest incremental prognostic information beyond conventional clinical variables. These findings suggest that dynamic CPP phenotyping may aid risk stratification and warrant further prospective validation.

## Data Availability

The datasets presented in this study can be found in online repositories. The names of the repository/repositories and accession number(s) can be found in the article/Supplementary material.
