# Supplementary material for: Cerebral perfusion pressure trajectories and cumulative exposure metrics predict in-hospital mortality in acute brain injury
Source: Front Med (Lausanne). 2026 May 22;13:1838528. doi: 10.3389/fmed.2026.1838528 (PMC13237695; doi:10.3389/fmed.2026.1838528)
Supplement: Supplementary file 1 [file Data_Sheet_1.docx]

Supplementary Figure 1. Flowchart of Participant Selection

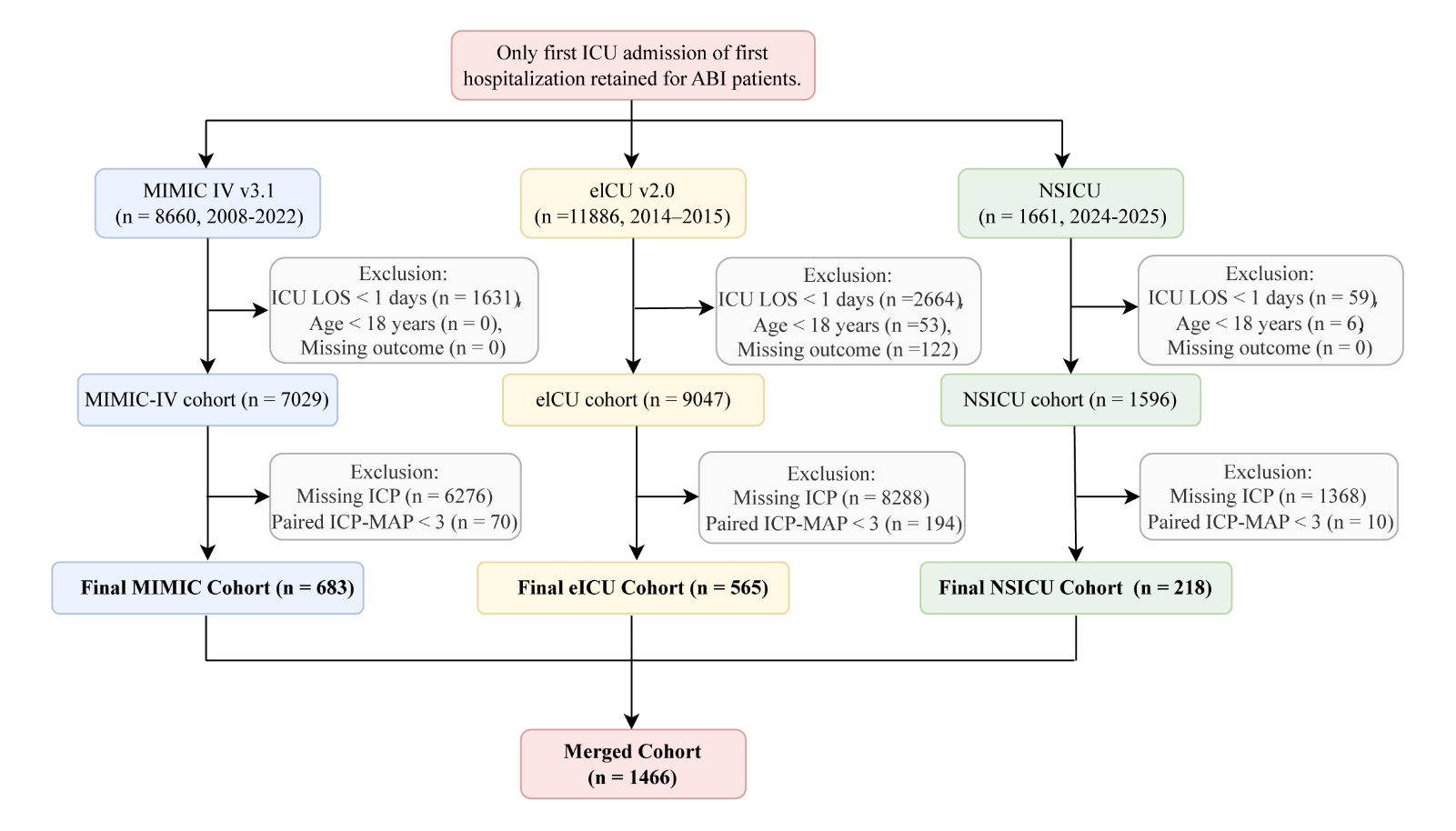

**Legend:** Flowchart of patient selection from the MIMIC-IV v3.1 (2008–2022), eICU v2.0 (2014–2015), and NSICU (2024–2025) databases. Only the first ICU admission during the first hospitalization was retained for ABI patients. Patients were excluded sequentially based on ICU length of stay (LOS <1 day), age (<18 years), missing outcome data, missing ICP measurements, or fewer than three valid paired ICP–MAP readings. The final merged cohort comprised 1,466 patients. Abbreviations: ABI, acute brain injury; ICP, intracranial pressure; MAP, mean arterial pressure; ICU, intensive care unit; LOS, length of stay.

# Supplementary Table 1. Clinical Variables Extracted for Analysis

| **Category** | **Variables** |
| --- | --- |
| **Demographic** | Age; sex (0 = Female, 1 = Male); body mass index (BMI, kg/m²); traumatic brain injury (TBI; 0 = No, 1 = Yes); admission type (0 = Elective, 1 = Emergency); admission time (0 = Day shift, 1 = Night shift); acute brain injury (ABI) subtype: acute ischemic stroke (AIS), intracerebral hemorrhage (ICH), subarachnoid hemorrhage (SAH), traumatic brain injury (TBI) |
| **Physiological Parameters** | Respiratory rate (bpm); heart rate (bpm); mean arterial pressure (MAP, mmHg); temperature (°C); initial intracranial pressure (ICP, mmHg); mean ICP (mmHg); final ICP (mmHg); baseline cerebral perfusion pressure (CPP, mmHg); mean CPP (mmHg); final CPP (mmHg); initial Glasgow Coma Scale (GCS) score; mean GCS score; final GCS score |
| **Laboratory Variables** | Urine output (mL); alanine aminotransferase (ALT, U/L); blood urea nitrogen (BUN, mg/dL); creatinine (mg/dL); glucose (mg/dL); red blood cell count (RBC, ×10¹²/L); white blood cell count (WBC, ×10⁹/L); platelet count (×10⁹/L); international normalized ratio (INR); total bilirubin (mg/dL); albumin (g/dL); sodium (mmol/L); potassium (mmol/L) |
| **Comorbidities and severity scores** | Hypertension (0 = No, 1 = Yes); diabetes (0 = No, 1 = Yes); history of stroke (0 = No, 1 = Yes); liver disease (0 = No, 1 = Yes); Charlson Comorbidity Index (CCI); Sequential Organ Failure Assessment (SOFA) score; Acute Physiology and Chronic Health Evaluation III (APACHE III) score |
| **Treatment Interventions** | Dialysis (0 = No, 1 = Yes); vasopressor use (0 = No, 1 = Yes); mannitol use (0 = No, 1 = Yes); mechanical ventilation (0 = No, 1 = Yes); craniotomy (0 = No, 1 = Yes); embolization (0 = No, 1 = Yes) |
| **Outcomes** | Discharge location: acute/ICU care (0 = No, 1 = Yes), chronic/long-term care (0 = No, 1 = Yes), home care/recovery (0 = No, 1 = Yes); length of hospital stay (days); length of ICU stay (days); in-hospital mortality (0 = No, 1 = Yes) |

# Supplementary Table 2. Missingness of Baseline Covariates in the Study Cohort

| **Variable** | **Missing, n** | **Missing, %** |
| --- | --- | --- |
| **ALT, U/L** | 635 | 43.3 |
| **SOFA score** | 332 | 22.6 |
| **APACHE III score** | 254 | 17.3 |
| **Urine output, mL** | 226 | 15.4 |
| **Charlson Comorbidity Index** | 218 | 14.9 |
| **BMI, kg/m²** | 205 | 14.0 |
| **INR** | 80 | 5.5 |
| **RBC count, ×10¹²/L** | 28 | 1.9 |
| **Platelet count, ×10⁹/L** | 26 | 1.8 |
| **Glucose, mg/dL** | 23 | 1.6 |
| **WBC count, ×10⁹/L** | 23 | 1.6 |
| **BUN, mg/dL** | 21 | 1.4 |
| **Creatinine, mg/dL** | 21 | 1.4 |
| **Potassium, mmol/L** | 21 | 1.4 |
| **Sodium, mmol/L** | 20 | 1.4 |
| **Temperature, °C** | 11 | 0.8 |
| **Heart rate, bpm** | 1 | 0.1 |
| **MAP, mmHg** | 1 | 0.1 |
| **Respiratory rate, breaths/min** | 1 | 0.1 |

**Notes:** Missingness is reported as the number (n) and percentage (%) of patients with missing values for each baseline covariate before imputation. Covariates not shown had 0% missingness.

# Supplementary Figure 2. Distributions of Observed and Imputed Values for Incomplete Covariates


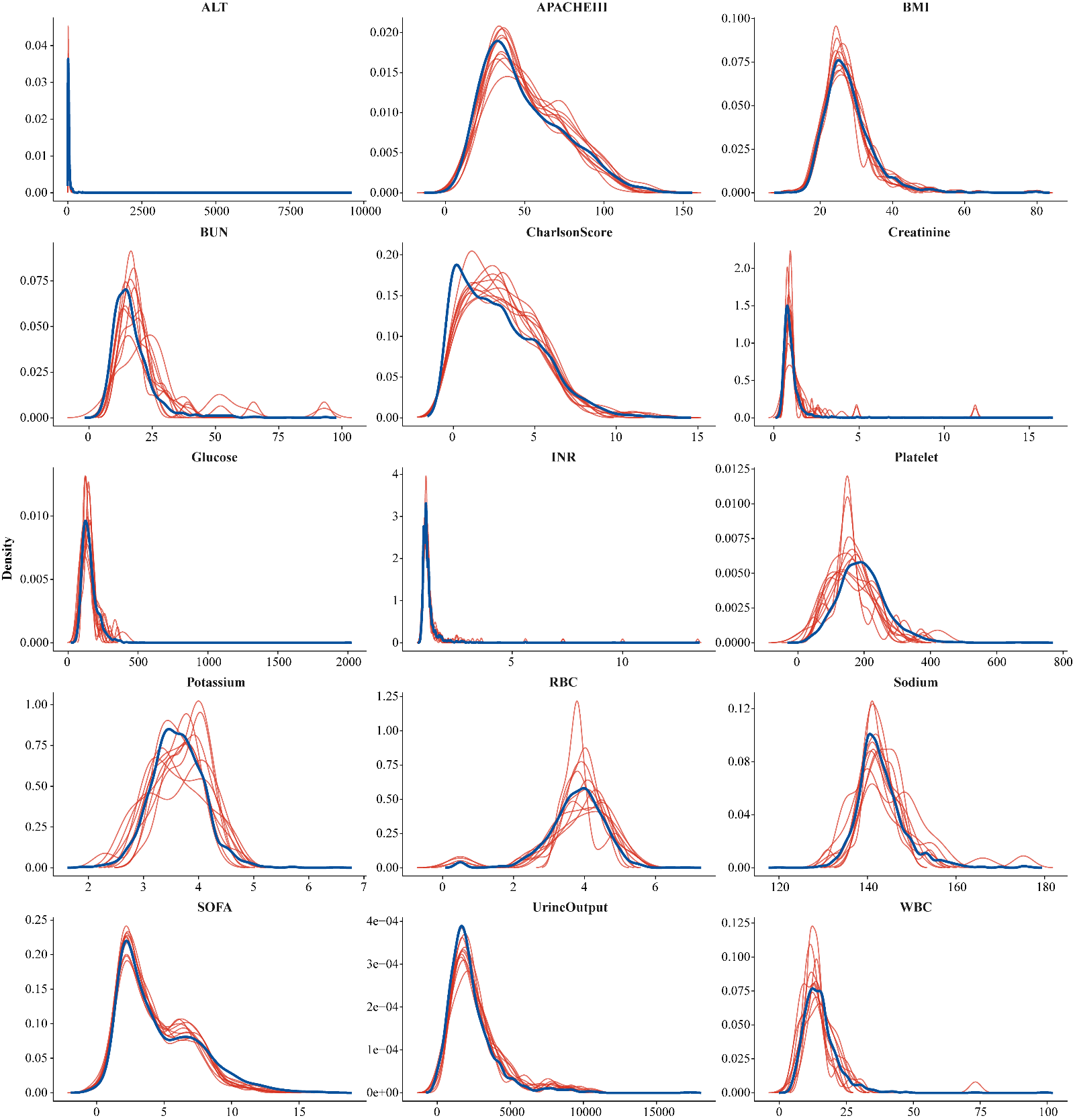


**Legend:** Kernel density plots comparing observed values (blue) with imputed values for missing entries only (red; m = 10 imputed datasets) for selected covariates with missing data. Each panel represents one variable.

# Supplementary Figure 3. Distributions of Observed and Completed Imputed Values for Incomplete Covariates


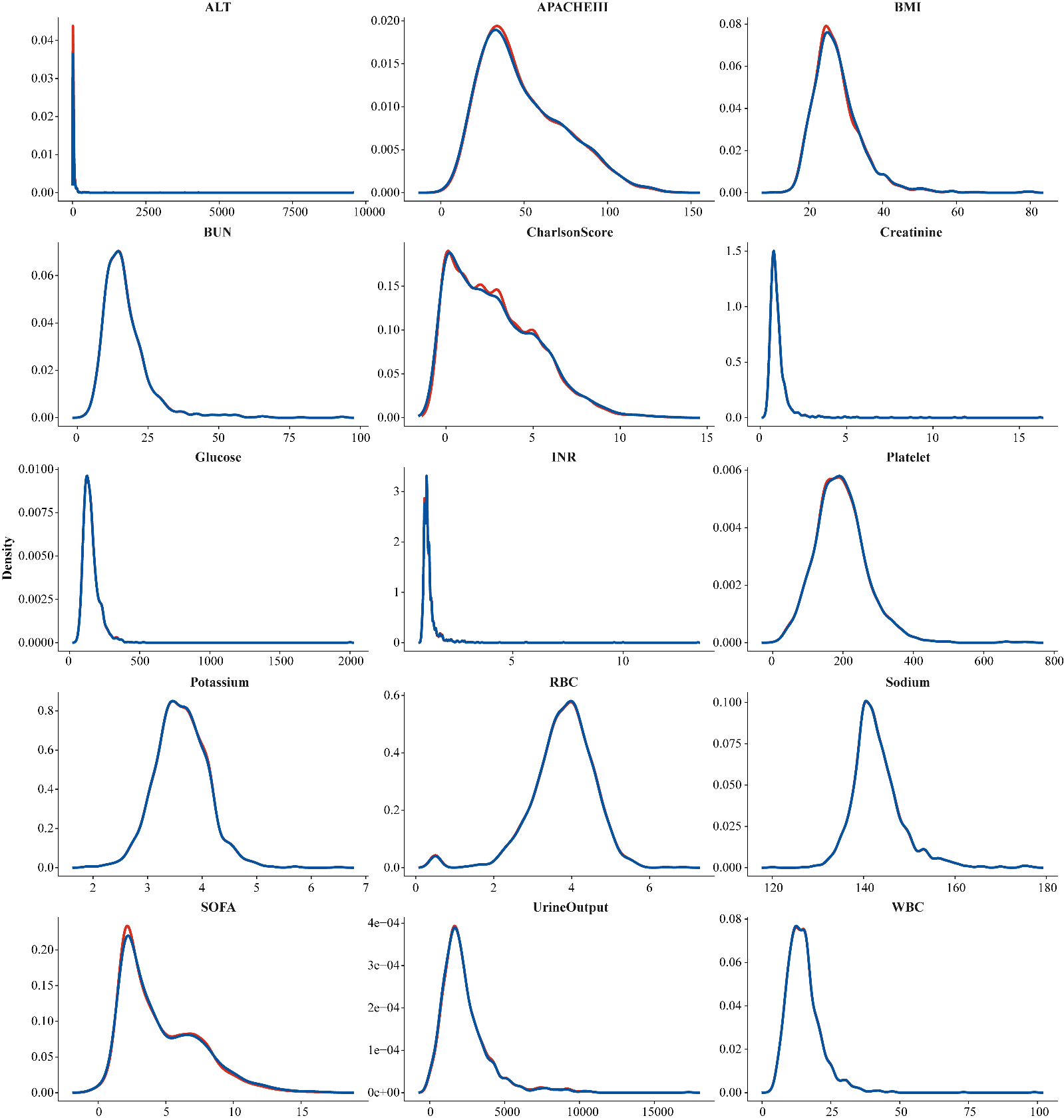


**Legend:** Kernel density plots comparing observed values (blue) with the corresponding distributions from completed imputed datasets (red; observed + imputed; m = 10) for selected covariates with missing data. Each panel represents one variable.

# Supplementary Figure 4. Trace Plots for Imputed Covariates


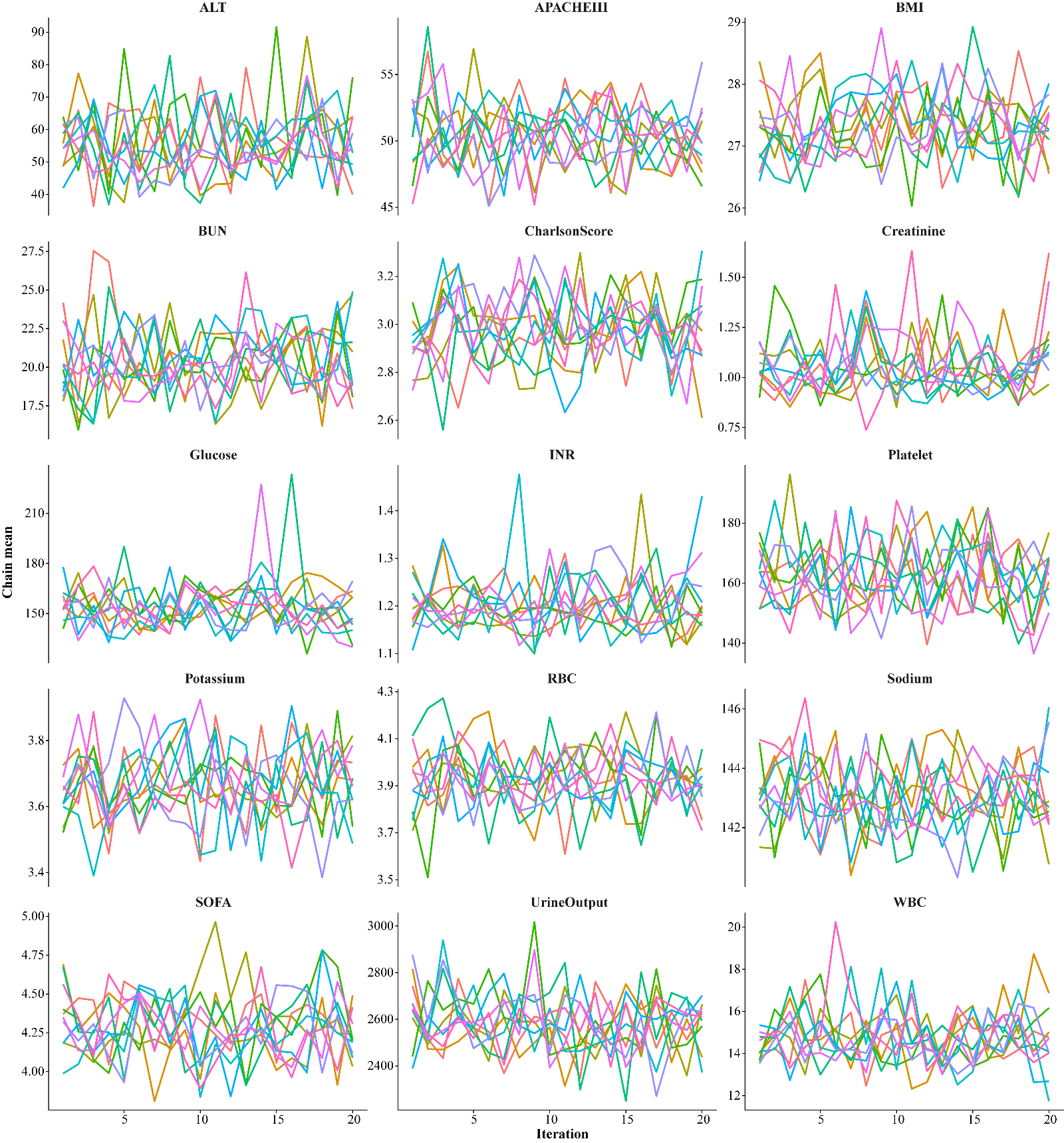


**Legend:** Trace plots of chain means across 20 iterations for selected covariates with missing data in the 10 imputed datasets. Each panel represents one variable, and each colored line represents one imputed dataset. These plots were used to assess convergence and stability of the multiple imputation procedure.

# Supplementary Figure 5. Distribution and Temporal Availability of Valid CPP Observations


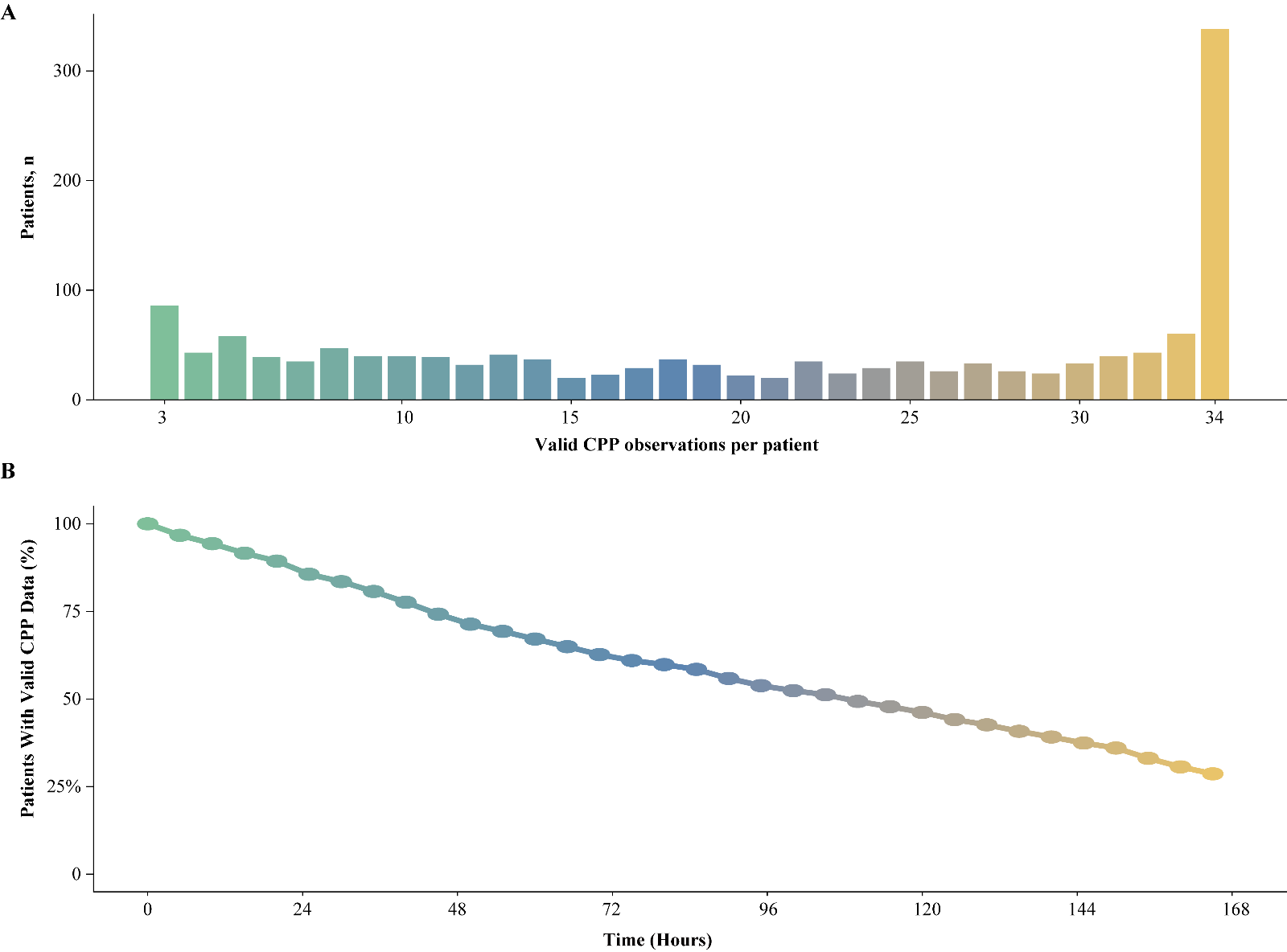

**Legend:** Panel A shows the distribution of the number of valid CPP observations per patient during the first 168 hours after ICU admission. Panel B shows the proportion of patients with valid CPP data at each 5-hour time point over the same period.

# Supplementary Figure 6. Illustration of Threshold-Specific CumCPP and ME-CumCPP


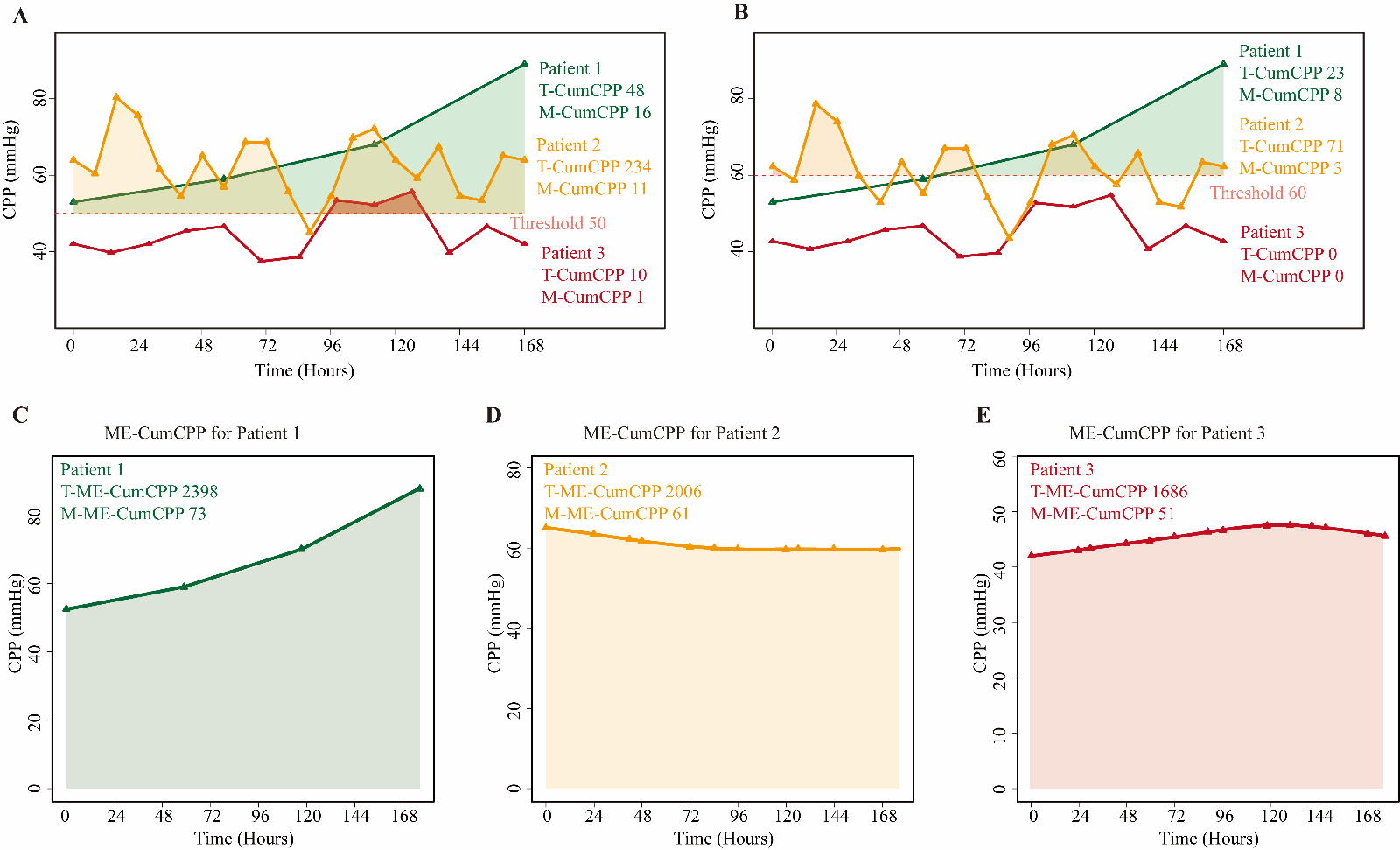

**Legend:** Panels A and B illustrate threshold-specific cumulative CPP at thresholds of 50 and 60 mmHg, respectively. Panels C–E illustrate mixed-effects–estimated cumulative CPP based on smoothed individual CPP profiles. Shaded regions represent longitudinal integration of CPP over time. Abbreviations: T-CumCPP-50/60, total threshold-specific cumulative CPP at thresholds of 50/60 mmHg; M-CumCPP-50/60, mean threshold-specific cumulative CPP at thresholds of 50/60 mmHg; T-ME-CumCPP, total mixed-effects–estimated cumulative CPP; M-ME-CumCPP, mean mixed-effects–estimated cumulative CPP.

# Supplementary Table 3. Model Fit Indices for CPP Trajectories in the Entire ABI Cohort

| **G** | **log-likelihood** | **AIC** | **BIC** | **SABIC** | **Entropy** | **Class, %** | | | | | | **Posterior probability** | | | | | |
| --- | --- | --- | --- | --- | --- | --- | --- | --- | --- | --- | --- | --- | --- | --- | --- | --- | --- |
|  |  |  |  |  |  | **1** | **2** | **3** | **4** | **5** | **6** | **1** | **2** | **3** | **4** | **5** | **6** |
| 2 | -117252.10 | 234534.19 | 234613.55 | 234565.90 | 0.87 | 5.73 | 94.27 |  |  |  |  | 0.86 | 0.98 |  |  |  |  |
| 3 | -117228.57 | 234497.15 | 234602.95 | 234539.42 | 0.48 | 4.98 | 73.74 | 21.28 |  |  |  | 0.80 | 0.74 | 0.70 |  |  |  |
| 4 | -117200.94 | 234451.88 | 234584.14 | 234504.72 | 0.47 | 28.72 | 35.06 | 29.95 | 6.28 |  |  | 0.73 | 0.61 | 0.69 | 0.84 |  |  |
| 5 | -117164.46 | 234388.92 | 234547.63 | 234452.33 | 0.52 | 2.18 | 32.20 | 34.45 | 5.66 | 25.51 |  | 0.80 | 0.61 | 0.71 | 0.77 | 0.67 |  |
| 6 | -117121.67 | 234313.35 | 234498.51 | 234387.33 | 0.61 | 2.25 | 1.98 | 29.13 | 48.16 | 13.92 | 4.57 | 0.77 | 0.80 | 0.70 | 0.74 | 0.59 | 0.74 |

**Note:** G indicates the number of latent classes. Model fit was evaluated using the log-likelihood, Akaike information criterion (AIC), Bayesian information criterion (BIC), sample-size adjusted BIC (SABIC), entropy, class proportions, and mean posterior probabilities. Higher entropy indicates better classification precision. Class percentages denote the proportion assigned to each latent class, and posterior probabilities denote the mean probability of assignment to the corresponding class. Although models with more classes yielded lower information criteria, the four-class solution was retained as the primary model based on overall balance among statistical fit, class distribution, posterior classification probabilities, and clinical interpretability.

# Supplementary Figure 7. Sensitivity Analyses of CPP Trajectory Phenotypes Using 3-Day and 5-Day Observation Windows


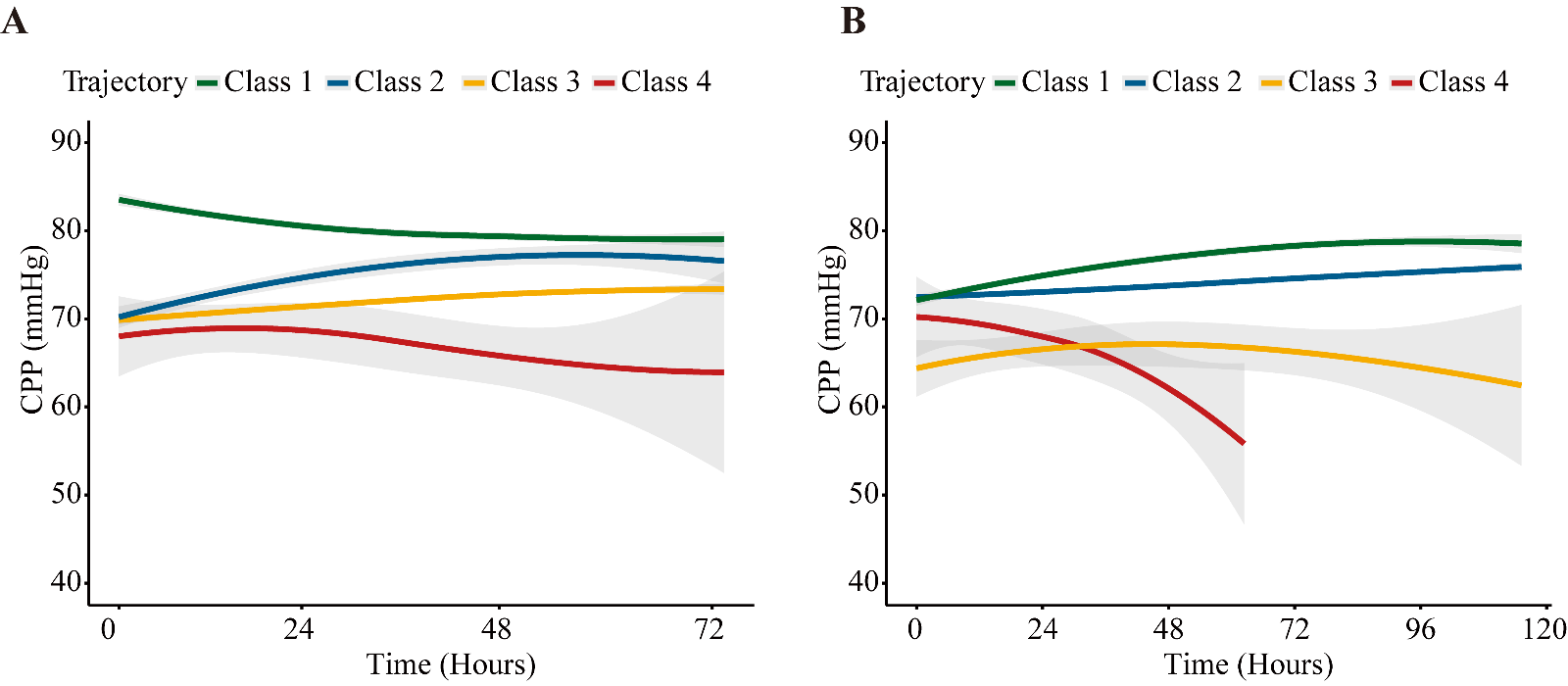

**Legend:** Modeled CPP trajectories with 95% confidence intervals (shaded regions) under alternative time-window/binning specifications. Panel A shows the four-class solution for the first 72 hours using 4-hour bins; Panel B shows the corresponding solution for the first 120 hours using 8-hour bins. Overall trajectory patterns remained broadly consistent.

# Supplementary Figure 8. Distribution of CPP Trajectory Phenotypes and In-Hospital Mortality by ABI Subtype and Data Source


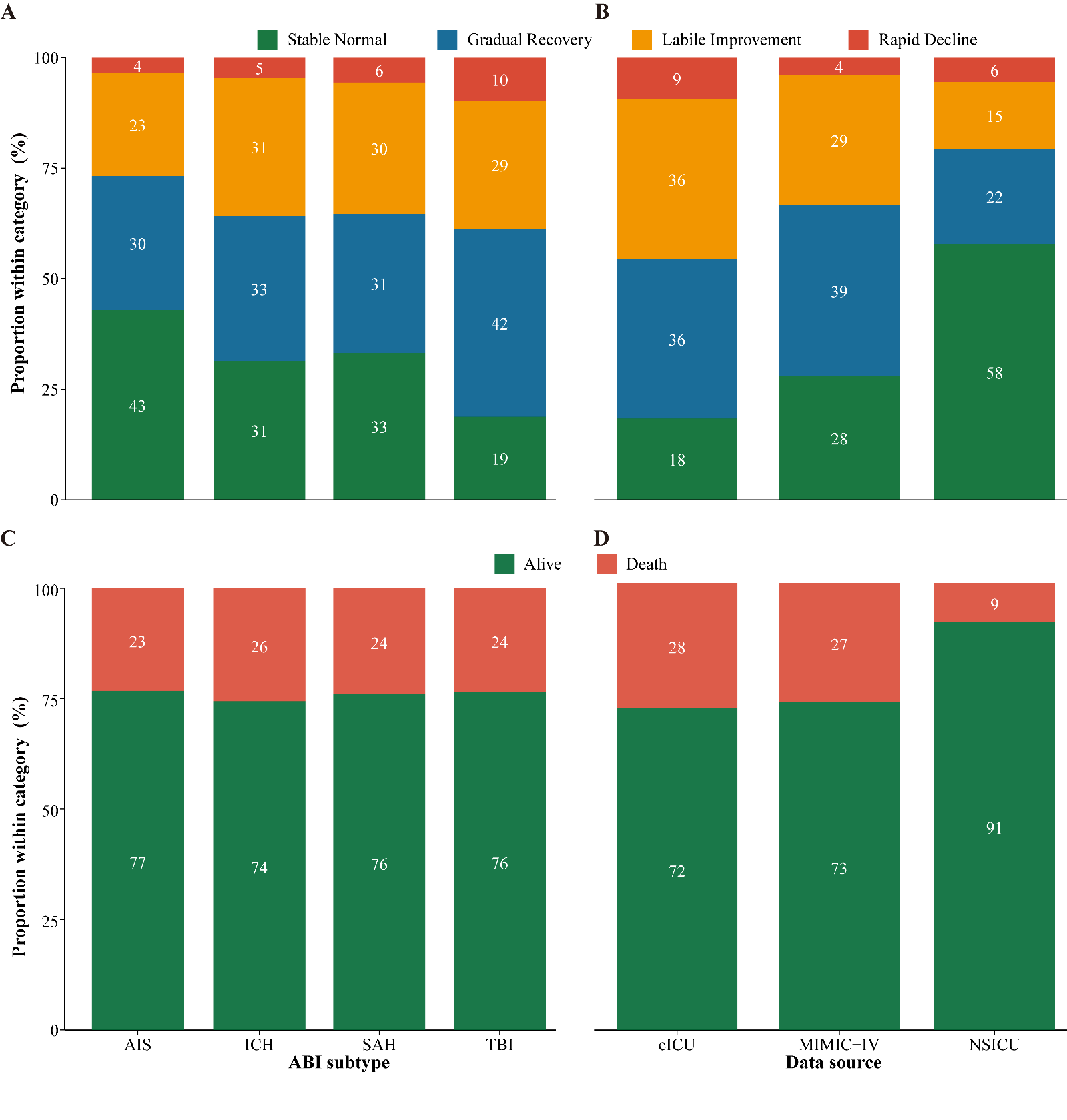
**Legend:** Panel A shows the distribution of CPP trajectory phenotypes across ABI subtypes (AIS, ICH, SAH, and TBI). Panel B shows the distribution of CPP trajectory phenotypes across data sources (eICU, MIMIC-IV, and NSICU). Panel C shows the distribution of in-hospital mortality across ABI subtypes, and Panel D shows the distribution of in-hospital mortality across data sources. Values within bars indicate the proportion within each category. Abbreviations: ABI, acute brain injury; AIS, acute ischemic stroke; ICH, intracerebral hemorrhage; SAH, subarachnoid hemorrhage; TBI, traumatic brain injury.

# Supplementary Figure 9. Clinical Profiles by CPP Trajectory Phenotypes


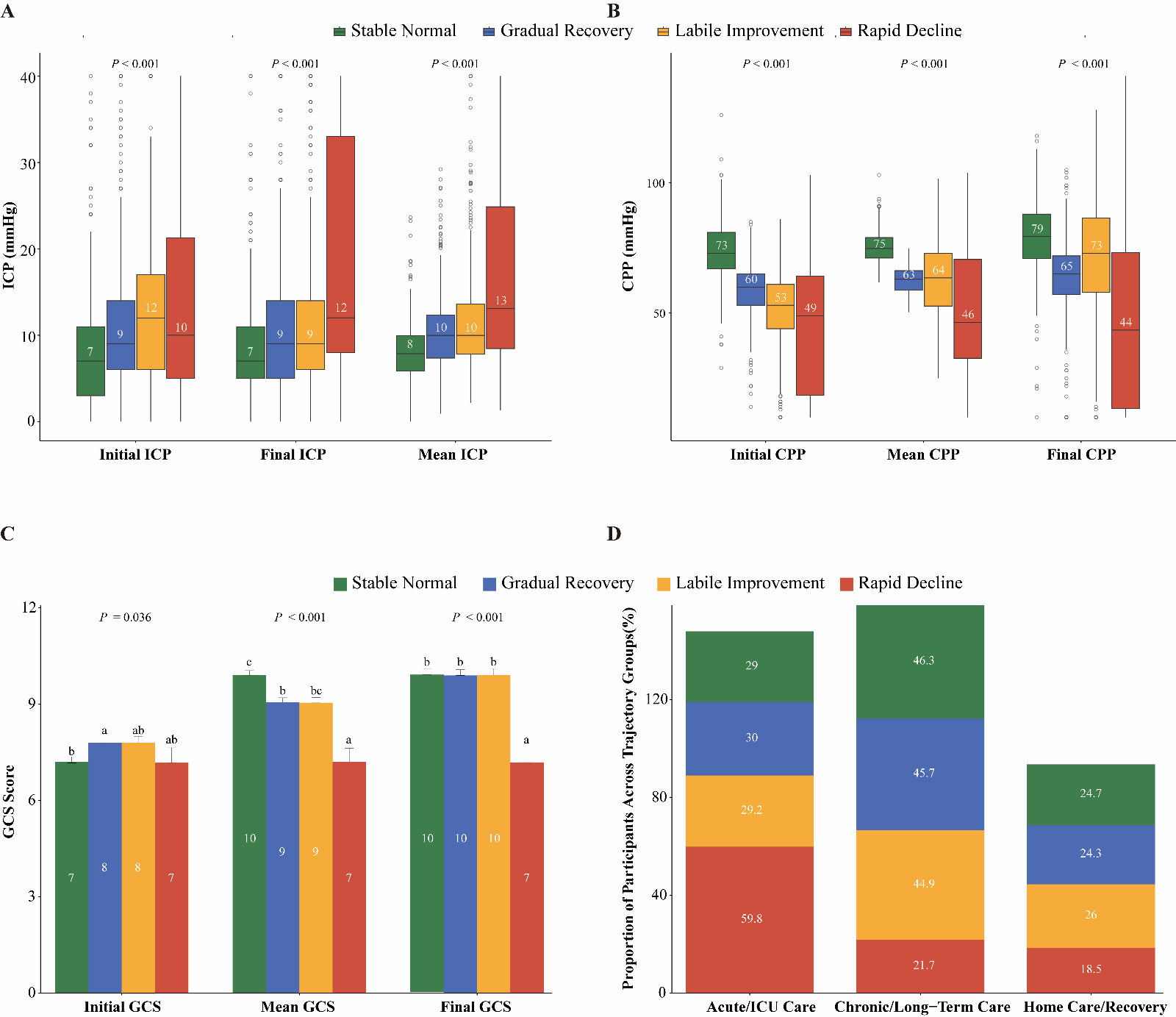


**Legend:** Panel A compares initial, final, and mean ICP values; Panel B compares initial, mean, and final CPP values; Panel C compares initial, mean, and final GCS scores, with lowercase letters indicating pairwise group differences; Panel D shows the proportions of discharge locations across trajectory phenotypes. *P* values represent overall comparisons across groups.

# Supplementary Table 4. Covariate Screening for Multivariable Models Using Change-in-Estimate and Collinearity Diagnostics

| **Variable** | **Base model** | | **Full model** | | **VIF** | **CIE ≥10%** | **Retained** |
| --- | --- | --- | --- | --- | --- | --- | --- |
|  | **HR** | **CIE (%)** | **HR** | **CIE (%)** |  |  |  |
| **Reference (GR)** | 0.68 | Ref. | 0.59 | Ref. | 1.464 | Ref. | Ref. |
| **Reference (LI)** | 0.76 | Ref. | 0.83 | Ref. | 1.464 | Ref. | Ref. |
| **Reference (RD)** | 1.81 | Ref. | 1.67 | Ref. | 1.464 | Ref. | Ref. |
| **Age (GR)** | 0.7 | 2.7 | 0.64 | 7.3 | 1.9 | No | No |
| **Age (LI)** | 0.81 | 6.9 | 0.79 | -4.8 | 1.9 | No | No |
| **Age (RD)** | 1.92 | 6.2 | 1.7 | 1.7 | 1.9 | No | No |
| **Sex (GR)** | 0.68 | -0.8 | 0.59 | 0.3 | 1.263 | No | No |
| **Sex (LI)** | 0.76 | 0.3 | 0.82 | -0.4 | 1.263 | No | No |
| **Sex (RD)** | 1.82 | 0.4 | 1.67 | -0.3 | 1.263 | No | No |
| **BMI (GR)** | 0.68 | -1 | 0.59 | -0.1 | 1.209 | No | No |
| **BMI (LI)** | 0.76 | 1 | 0.8 | -4 | 1.209 | No | No |
| **BMI (RD)** | 1.82 | 0.8 | 1.63 | -2.3 | 1.209 | No | No |
| **TBI (GR)** | 0.72 | 5 | 0.57 | -4.1 | 1.668 | No | No |
| **TBI (LI)** | 0.77 | 2.2 | 0.8 | -3.1 | 1.668 | No | No |
| **TBI (RD)** | 1.86 | 2.6 | 1.64 | -1.7 | 1.668 | No | No |
| **Admission Type (GR)** | 0.67 | -1.6 | 0.59 | 0 | 1.244 | No | No |
| **Admission Type (LI)** | 0.74 | -1.4 | 0.83 | 0 | 1.244 | No | No |
| **Admission Type (RD)** | 1.79 | -1.4 | 1.68 | 0.5 | 1.244 | No | No |
| **Admission Time (GR)** | 0.68 | -0.7 | 0.59 | 0.4 | 1.11 | No | No |
| **Admission Time (LI)** | 0.75 | -0.8 | 0.83 | -0.2 | 1.11 | No | No |
| **Admission Time (RD)** | 1.81 | 0.2 | 1.67 | -0.2 | 1.11 | No | No |
| **Respiratory Rate (GR)** | 0.67 | -1.5 | 0.59 | 0.2 | 1.266 | No | No |
| **Respiratory Rate (LI)** | 0.75 | -1.3 | 0.83 | 0.2 | 1.266 | No | No |
| **Respiratory Rate (RD)** | 1.79 | -1.4 | 1.68 | 0.2 | 1.266 | No | No |
| **Heart Rate (GR)** | 0.68 | -0.7 | 0.57 | -4.6 | 1.756 | No | No |
| **Heart Rate (LI)** | 0.74 | -1.4 | 0.81 | -2 | 1.756 | No | No |
| **Heart Rate (RD)** | 1.77 | -2 | 1.66 | -0.5 | 1.756 | No | No |
| **MAP (GR)** | 0.69 | 0.5 | 0.59 | -0.6 | 1.243 | No | No |
| **MAP (LI)** | 0.76 | 0.6 | 0.82 | -0.5 | 1.243 | No | No |
| **MAP (RD)** | 1.81 | 0 | 1.67 | -0.1 | 1.243 | No | No |
| **Temperature (GR)** | 0.68 | -0.9 | 0.59 | -0.3 | 1.214 | No | No |
| **Temperature (LI)** | 0.74 | -1.9 | 0.85 | 2.1 | 1.214 | No | No |
| **Temperature (RD)** | 1.76 | -2.9 | 1.7 | 1.5 | 1.214 | No | No |
| **Urine Output (GR)** | 0.65 | -4.5 | 0.61 | 2.5 | 1.762 | No | No |
| **Urine Output (LI)** | 0.72 | -4.1 | 0.85 | 2.5 | 1.762 | No | No |
| **Urine Output (RD)** | 1.7 | -6.1 | 1.78 | 6.5 | 1.762 | No | No |
| **ALT (GR)** | 0.68 | -0.2 | 0.6 | 0.6 | 1.917 | No | No |
| **ALT (LI)** | 0.75 | -1.1 | 0.83 | 0.4 | 1.917 | No | No |
| **ALT (RD)** | 1.81 | 0 | 1.67 | -0.2 | 1.917 | No | No |
| **BUN (GR)** | 0.67 | -2.2 | 0.59 | 0.1 | 2.394 | No | No |
| **BUN (LI)** | 0.76 | 1.1 | 0.83 | 0 | 2.394 | No | No |
| **BUN (RD)** | 1.86 | 2.6 | 1.67 | -0.1 | 2.394 | No | No |
| **Creatinine (GR)** | 0.68 | -1.2 | 0.6 | 0.5 | 2.593 | No | No |
| **Creatinine (LI)** | 0.75 | -1.3 | 0.84 | 2 | 2.593 | No | No |
| **Creatinine (RD)** | 1.74 | -3.9 | 1.69 | 1.4 | 2.593 | No | No |
| **Glucose (GR)** | 0.64 | -6.3 | 0.62 | 4.5 | 1.146 | No | No |
| **Glucose (LI)** | 0.74 | -1.7 | 0.84 | 1 | 1.146 | No | No |
| **Glucose (RD)** | 1.8 | -0.4 | 1.67 | 0.2 | 1.146 | No | No |
| **Sodium (GR)** | 0.62 | -9.1 | 0.63 | 5.7 | 1.684 | No | No |
| **Sodium (LI)** | 0.73 | -3.2 | 0.82 | -0.7 | 1.684 | No | No |
| **Sodium (RD)** | 1.65 | -9 | 1.69 | 1.2 | 1.684 | No | No |
| **Potassium (GR)** | 0.69 | 1 | 0.57 | -3.8 | 1.498 | No | No |
| **Potassium (LI)** | 0.76 | 0.2 | 0.84 | 1.1 | 1.498 | No | No |
| **Potassium (RD)** | 1.84 | 1.4 | 1.64 | -1.6 | 1.498 | No | No |
| **RBC (GR)** | 0.67 | -1.9 | 0.59 | 0.1 | 1.387 | No | No |
| **RBC (LI)** | 0.75 | -1.3 | 0.83 | 0 | 1.387 | No | No |
| **RBC (RD)** | 1.79 | -1.3 | 1.67 | 0.1 | 1.387 | No | No |
| **WBC (GR)** | 0.66 | -3.1 | 0.59 | -0.3 | 1.298 | No | No |
| **WBC (LI)** | 0.71 | -5.6 | 0.83 | 0.7 | 1.298 | No | No |
| **WBC (RD)** | 1.75 | -3.5 | 1.69 | 1.3 | 1.298 | No | No |
| **Platelet (GR)** | 0.67 | -1.8 | 0.59 | 0.1 | 1.399 | No | No |
| **Platelet (LI)** | 0.75 | -0.4 | 0.82 | -0.6 | 1.399 | No | No |
| **Platelet (RD)** | 1.81 | 0.2 | 1.66 | -0.6 | 1.399 | No | No |
| **INR (GR)** | 0.69 | 1 | 0.59 | -0.1 | 1.827 | No | No |
| **INR (LI)** | 0.76 | 0.7 | 0.83 | 0.1 | 1.827 | No | No |
| **INR (RD)** | 1.81 | 0.2 | 1.67 | 0 | 1.827 | No | No |
| **Hypertension (GR)** | 0.75 | 10.4 | 0.55 | -7 | 1.53 | Yes | Yes |
| **Hypertension (LI)** | 0.83 | 9.7 | 0.81 | -2.7 | 1.53 | No | No |
| **Hypertension (RD)** | 1.89 | 4.7 | 1.65 | -1 | 1.53 | No | No |
| **Diabetes (GR)** | 0.67 | -2.3 | 0.58 | -1.4 | 1.227 | No | No |
| **Diabetes (LI)** | 0.76 | 0.2 | 0.82 | -0.7 | 1.227 | No | No |
| **Diabetes (RD)** | 1.81 | 0.2 | 1.66 | -0.4 | 1.227 | No | No |
| **Stroke History (GR)** | 0.68 | -0.9 | 0.6 | 1.5 | 1.17 | No | No |
| **Stroke History (LI)** | 0.75 | -1.1 | 0.84 | 1.8 | 1.17 | No | No |
| **Stroke History (RD)** | 1.81 | 0 | 1.67 | -0.1 | 1.17 | No | No |
| **Liver Disease (GR)** | 0.69 | 0.9 | 0.59 | -0.4 | 1.11 | No | No |
| **Liver Disease (LI)** | 0.76 | 0.7 | 0.83 | 0 | 1.11 | No | No |
| **Liver Disease (RD)** | 1.81 | 0.2 | 1.67 | 0.2 | 1.11 | No | No |
| **Initial GCS (GR)** | 0.69 | 1.6 | 0.59 | 0.3 | 1.355 | No | No |
| **Initial GCS (LI)** | 0.77 | 1.7 | 0.83 | 0.3 | 1.355 | No | No |
| **Initial GCS (RD)** | 1.82 | 0.4 | 1.67 | 0.1 | 1.355 | No | No |
| **Dialysis (GR)** | 0.67 | -1.3 | 0.61 | 3.3 | 1.694 | No | No |
| **Dialysis (LI)** | 0.75 | -0.4 | 0.83 | -0.3 | 1.694 | No | No |
| **Dialysis (RD)** | 1.78 | -1.8 | 1.69 | 1.1 | 1.694 | No | No |
| **Vasopressor (GR)** | 0.63 | -7.9 | 0.6 | 1.8 | 1.263 | No | No |
| **Vasopressor (LI)** | 0.68 | -9.7 | 0.84 | 1.9 | 1.263 | No | No |
| **Vasopressor (RD)** | 1.71 | -5.4 | 1.69 | 1 | 1.263 | No | No |
| **Mannitol (GR)** | 0.68 | -1.1 | 0.6 | 0.9 | 1.255 | No | No |
| **Mannitol (LI)** | 0.77 | 2.2 | 0.82 | -0.9 | 1.255 | No | No |
| **Mannitol (RD)** | 1.82 | 0.7 | 1.67 | -0.2 | 1.255 | No | No |
| **Ventilation (GR)** | 0.64 | -6.4 | 0.61 | 2.6 | 1.174 | No | No |
| **Ventilation (LI)** | 0.7 | -7.5 | 0.87 | 4.6 | 1.174 | No | No |
| **Ventilation (RD)** | 1.78 | -1.5 | 1.7 | 1.9 | 1.174 | No | No |
| **Craniotomy (GR)** | 0.68 | -0.2 | 0.59 | -1.1 | 1.147 | No | No |
| **Craniotomy (LI)** | 0.75 | -0.3 | 0.82 | -1.1 | 1.147 | No | No |
| **Craniotomy (RD)** | 1.81 | -0.3 | 1.67 | -0.1 | 1.147 | No | No |
| **Embolization (GR)** | 0.67 | -1.9 | 0.58 | -1.8 | 1.318 | No | No |
| **Embolization (LI)** | 0.8 | 6.2 | 0.77 | -6.9 | 1.318 | No | No |
| **Embolization (RD)** | 1.8 | -0.8 | 1.66 | -0.7 | 1.318 | No | No |

**Notes:** Covariates were screened using a change-in-estimate approach with collinearity diagnostics. In the Base model columns, HR denotes the hazard ratio for the trajectory phenotype after adding each candidate covariate individually to the base model, and CIE (%) denotes the corresponding percentage change relative to the base-model estimate. In the Full model columns, HR denotes the hazard ratio for the trajectory phenotype after removing each covariate one at a time from the prespecified full model, and CIE (%) denotes the corresponding percentage change relative to the full-model estimate. A prespecified threshold of absolute CIE ≥10% was used for screening, and collinearity was assessed using variance inflation factors (VIF). Only hypertension met the screening threshold. GR, Gradual Recovery; LI, Labile Improvement; RD, Rapid Decline; Stable Normal was the reference phenotype. Final covariate inclusion was determined primarily by prespecified clinical relevance, with statistical screening used as supplementary information; accordingly, age, sex, TBI, temperature, urine output, ALT, BUN, glucose, sodium, hypertension, diabetes, initial GCS, mannitol use, vasopressor use, ventilation, and craniotomy were retained in the final multivariable models.

# Supplementary Figure 10. Kaplan–Meier Survival Curves Stratified by Four CPP Trajectories


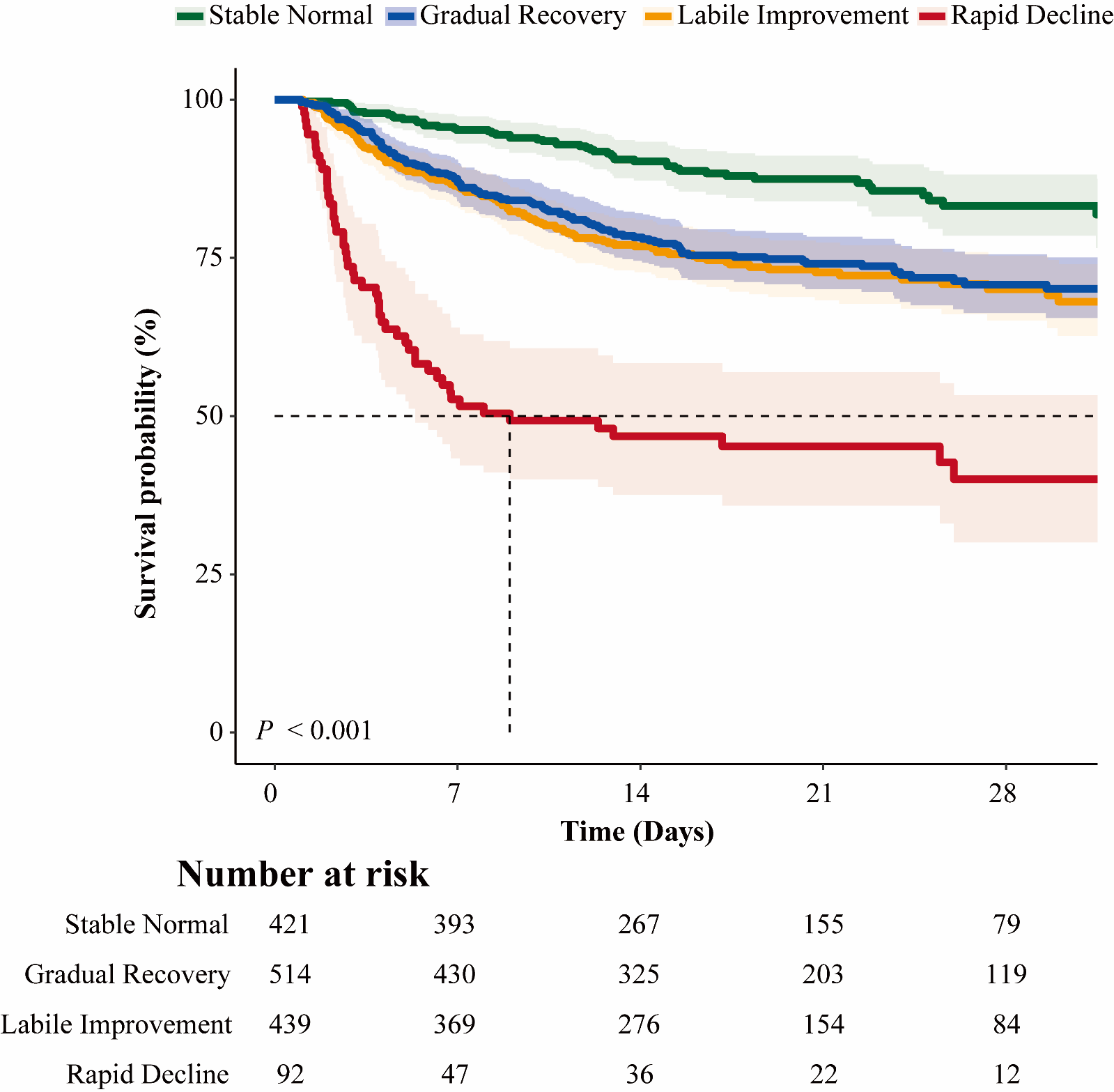


**Legend:** Shaded areas indicate 95% confidence intervals, and numbers at risk are shown at each time point. The horizontal dashed line marks the 50% survival probability, and the vertical dashed line indicates the median survival time for the Rapid Decline phenotype. Stable Normal showed the highest survival probability, whereas Rapid Decline showed the lowest.

# Supplementary Figure 11. E-values for CPP Trajectory Phenotypes and Baseline CPP


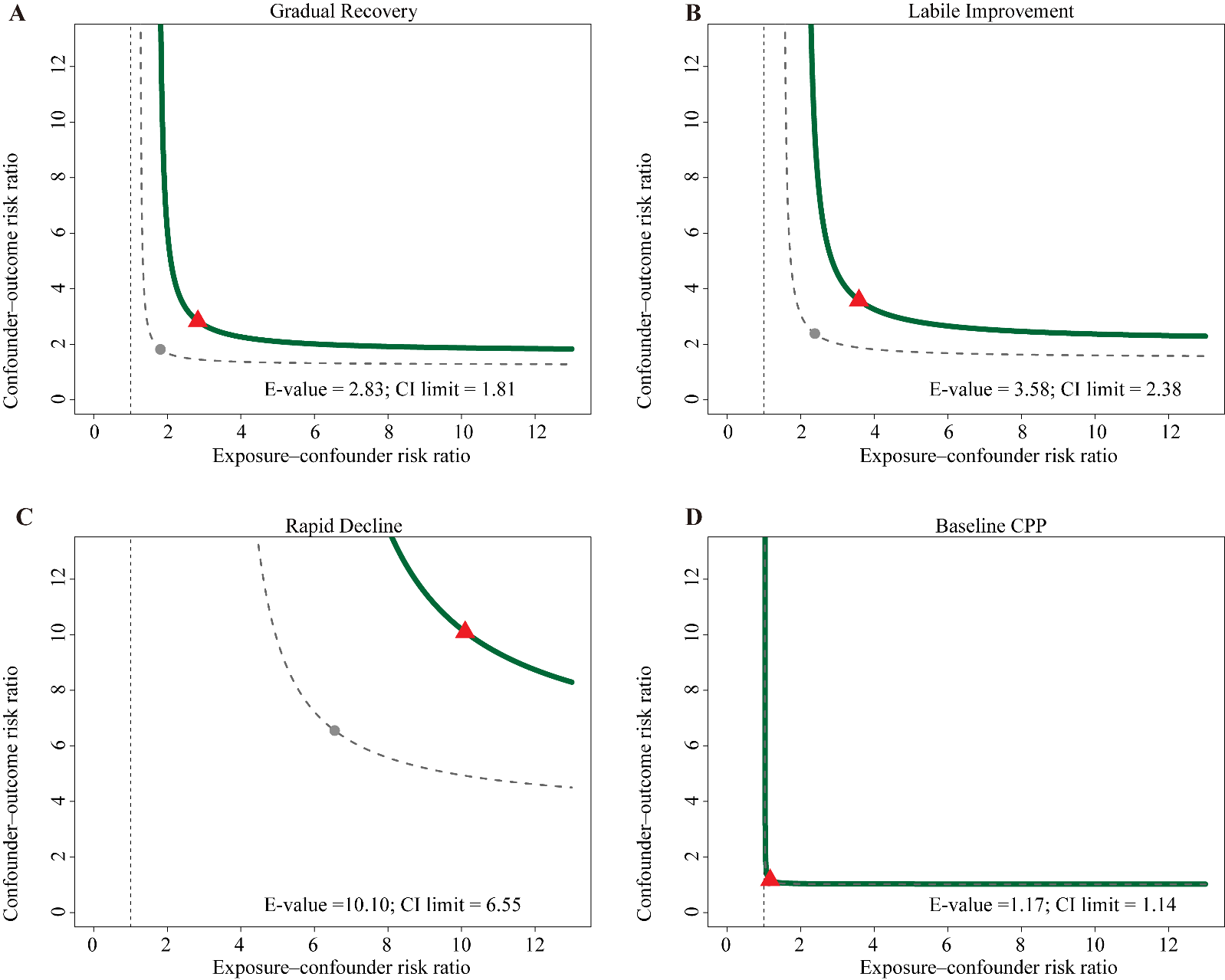


**Legend:** Panels A–C show E-values for the associations of Gradual Recovery, Labile Improvement, and Rapid Decline, respectively, with Stable Normal as the reference phenotype. Panel D shows the E-value for Baseline CPP. Red triangles indicate point estimates, and gray circles indicate the lower confidence-limit values used to calculate the corresponding E-values. Larger E-values indicate greater robustness to potential unmeasured confounding.

# Supplementary Table 5. Time-Stratified Cox Regression Analyses of CPP Trajectory Phenotypes and In-Hospital Mortality

| **Variable** | **Model 1** | | **Model 2** | | **Model 3** | |
| --- | --- | --- | --- | --- | --- | --- |
|  | **HR (95% CI)** | ***P*** | **HR (95% CI)** | ***P*** | **HR (95% CI)** | ***P*** |
| **Time interval: 0–3 days** | | | | | | |
| **Stable Normal** | Ref | — | Ref | — | Ref | — |
| **Gradual Recovery** | 1.98 (0.87–4.53) | 0.105 | 1.63 (0.71–3.73) | 0.249 | 1.61 (0.70–3.68) | 0.264 |
| **Labile Improvement** | 2.97 (1.33–6.60) | 0.008 | 2.82 (1.27–6.30) | 0.011 | 2.69 (1.20–6.00) | 0.016 |
| **Rapid Decline** | 16.95 (7.64–37.58) | <0.001 | 12.22 (5.42–27.58) | <0.001 | 11.39 (5.03–25.79) | <0.001 |
| **Time interval: >3 days** | | | | | | |
| **Stable Normal** | Ref | — | Ref | — | Ref | — |
| **Gradual Recovery** | 1.98 (1.42–2.77) | <0.001 | 1.77 (1.26–2.49) | <0.001 | 1.76 (1.25–2.48) | 0.001 |
| **Labile Improvement** | 1.99 (1.41–2.81) | <0.001 | 2.05 (1.45–2.90) | <0.001 | 1.98 (1.40–2.82) | <0.001 |
| **Rapid Decline** | 3.88 (2.41–6.24) | <0.001 | 3.73 (2.28–6.10) | <0.001 | 3.73 (2.28–6.10) | <0.001 |
| **Time interval: 0–5 days** | | | | | | |
| **Stable Normal** | Ref | — | Ref | — | Ref | — |
| **Gradual Recovery** | 3.20 (1.74–5.90) | <0.001 | 2.67 (1.45–4.94) | 0.002 | 2.64 (1.43–4.89) | 0.002 |
| **Labile Improvement** | 3.73 (2.02–6.88) | <0.001 | 3.60 (1.95–6.65) | <0.001 | 3.45 (1.86–6.40) | <0.001 |
| **Rapid Decline** | 16.08 (8.50–30.39) | <0.001 | 12.32 (6.40–23.74) | <0.001 | 11.81 (6.12–22.81) | <0.001 |
| **Time interval: >5 days** | | | | | | |
| **Stable Normal** | Ref | — | Ref | — | Ref | — |
| **Gradual Recovery** | 1.62 (1.12–2.33) | 0.010 | 1.47 (1.02–2.13) | 0.039 | 1.47 (1.01–2.12) | 0.043 |
| **Labile Improvement** | 1.66 (1.14–2.41) | 0.009 | 1.73 (1.18–2.53) | 0.005 | 1.67 (1.14–2.45) | 0.008 |
| **Rapid Decline** | 2.74 (1.54–4.86) | <0.001 | 2.79 (1.55–5.01) | <0.001 | 2.79 (1.55–5.02) | <0.001 |
| **Time interval: 0–3 days** | | | | | | |
| **Stable Normal** | Ref | — | Ref | — | Ref | — |
| **Gradual Recovery** | 1.98 (0.87–4.53) | 0.105 | 1.63 (0.71–3.74) | 0.246 | 1.61 (0.70–3.69) | 0.261 |
| **Labile Improvement** | 2.97 (1.33–6.60) | 0.008 | 2.83 (1.27–6.30) | 0.011 | 2.69 (1.21–6.01) | 0.016 |
| **Rapid Decline** | 16.95 (7.64–37.58) | <0.001 | 12.28 (5.44–27.70) | <0.001 | 11.45 (5.06–25.92) | <0.001 |
| **Time interval: 3–5 days** | | | | | | |
| **Stable Normal** | Ref | — | Ref | — | Ref | — |
| **Gradual Recovery** | 5.19 (2.01–13.37) | <0.001 | 4.40 (1.70–11.36) | 0.002 | 4.36 (1.69–11.26) | 0.002 |
| **Labile Improvement** | 4.95 (1.89–12.96) | 0.001 | 4.84 (1.84–12.69) | 0.001 | 4.68 (1.78–12.30) | 0.002 |
| **Rapid Decline** | 13.33 (4.55–38.99) | <0.001 | 11.00 (3.72–32.49) | <0.001 | 10.93 (3.70–32.32) | <0.001 |
| **Time interval: >5 days** | | | | | | |
| **Stable Normal** | Ref | — | Ref | — | Ref | — |
| **Gradual Recovery** | 1.62 (1.12–2.33) | 0.010 | 1.47 (1.02–2.13) | 0.039 | 1.47 (1.01–2.13) | 0.043 |
| **Labile Improvement** | 1.66 (1.14–2.41) | 0.009 | 1.73 (1.18–2.53) | 0.005 | 1.67 (1.14–2.45) | 0.008 |
| **Rapid Decline** | 2.74 (1.54–4.86) | <0.001 | 2.79 (1.55–5.01) | <0.001 | 2.80 (1.56–5.02) | <0.001 |

**Note:** Time-stratified Cox models were fitted within prespecified follow-up intervals. CPP trajectory phenotype was the exposure, with Stable Normal as the reference. Entries are HRs (95% CIs) with two-sided *P* values. Model 1 was unadjusted; Model 2 was adjusted for age, sex, TBI, temperature, urine output, ALT, BUN, glucose, and sodium; Model 3 was further adjusted for hypertension, diabetes, initial GCS, mannitol use, vasopressor use, ventilation, and craniotomy.

# Supplementary Figure 12. Forest Plots of Time-Stratified Associations Between CPP Trajectory Phenotypes and In-Hospital Mortality


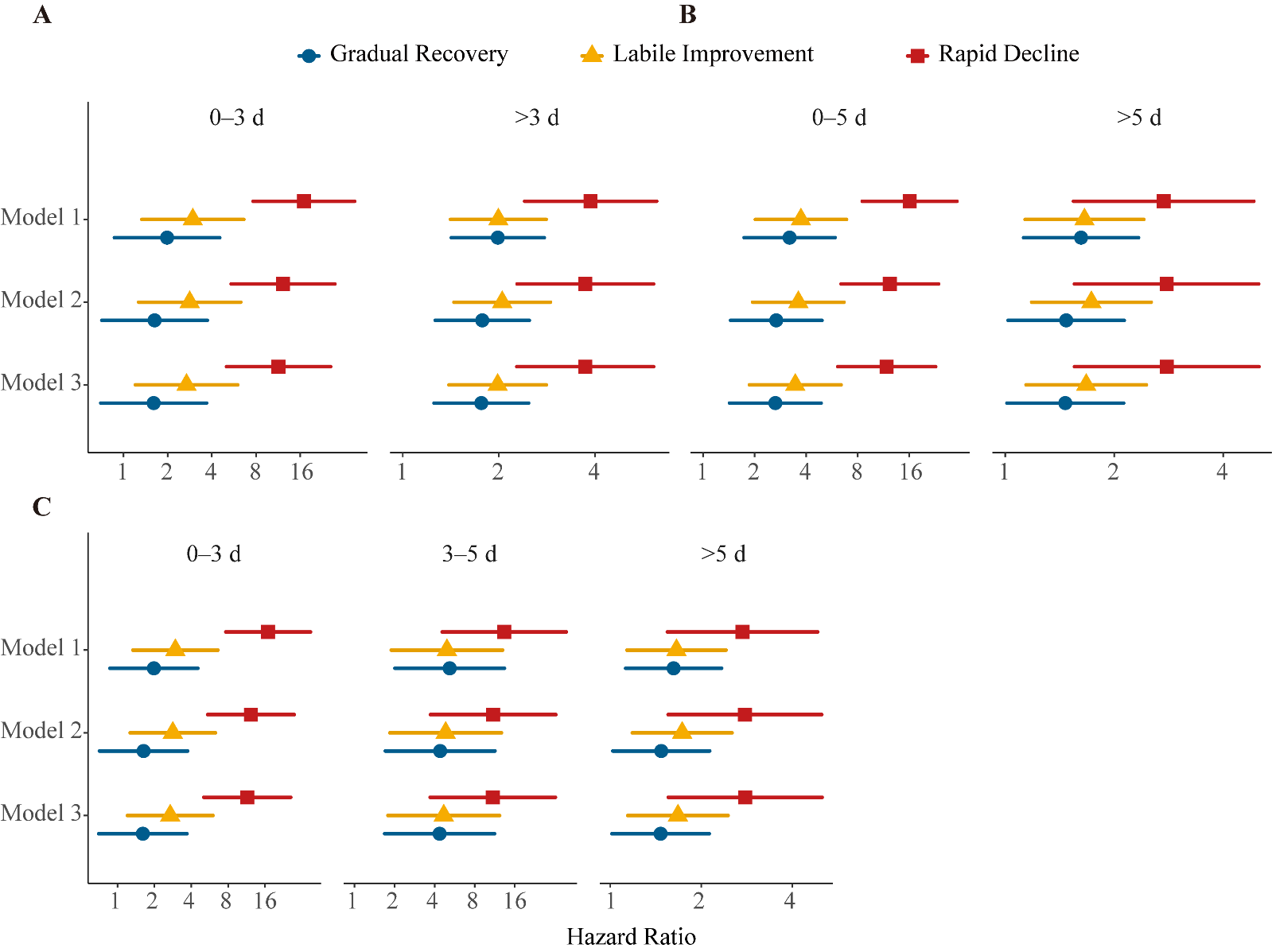


**Legend:** Forest plots of time-stratified Cox regression analyses for in-hospital mortality across CPP trajectory phenotypes, using Stable Normal as the reference. Panel A shows results for 0–3 versus >3 days, Panel B for 0–5 versus >5 days, and Panel C for 0–3, 3–5, and >5 days. Points indicate hazard ratios and horizontal lines indicate 95% confidence intervals for Gradual Recovery, Labile Improvement, and Rapid Decline. Model 1 was unadjusted; Model 2 adjusted for age, sex, TBI, temperature, urine output, ALT, BUN, glucose, and sodium; Model 3 further adjusted for hypertension, diabetes, initial GCS, mannitol use, vasopressor use, ventilation, and craniotomy.

# Supplementary Figure 13. Additional Subgroup Analysis of Mortality by CPP Trajectory Phenotypes


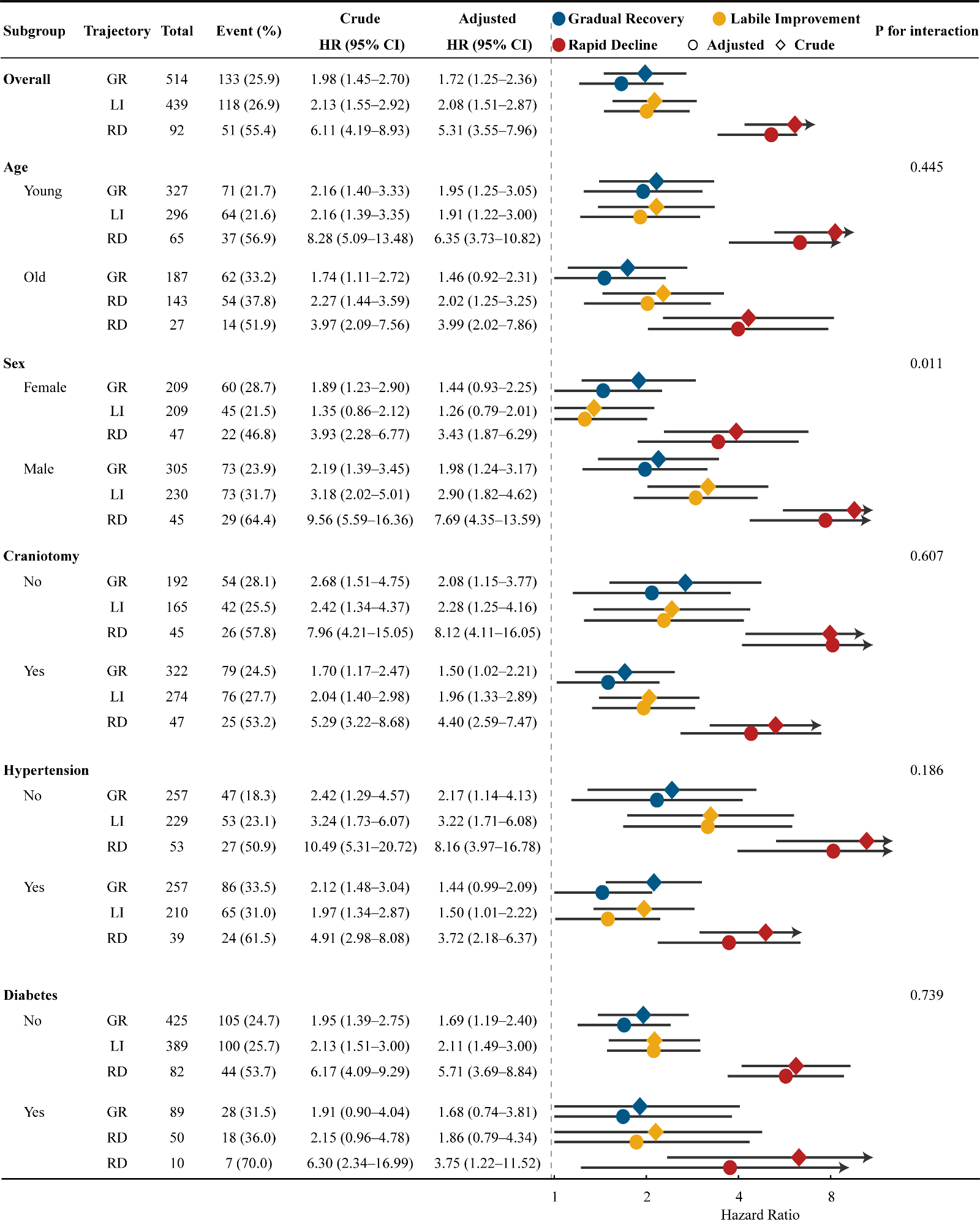


**Legend:** Subgroup analyses of in-hospital mortality across CPP trajectory phenotypes, with Stable Normal as the reference. Subgroups were defined by age, sex, craniotomy, hypertension, and diabetes. Diamonds indicate crude hazard ratios and circles indicate adjusted hazard ratios derived from the fully adjusted model; horizontal lines represent 95% confidence intervals. Interaction *P* values are shown for each subgroup category.

# Supplementary Figure 14. Variable Selection Patterns Under BSS Criteria for In-Hospital Mortality Prediction


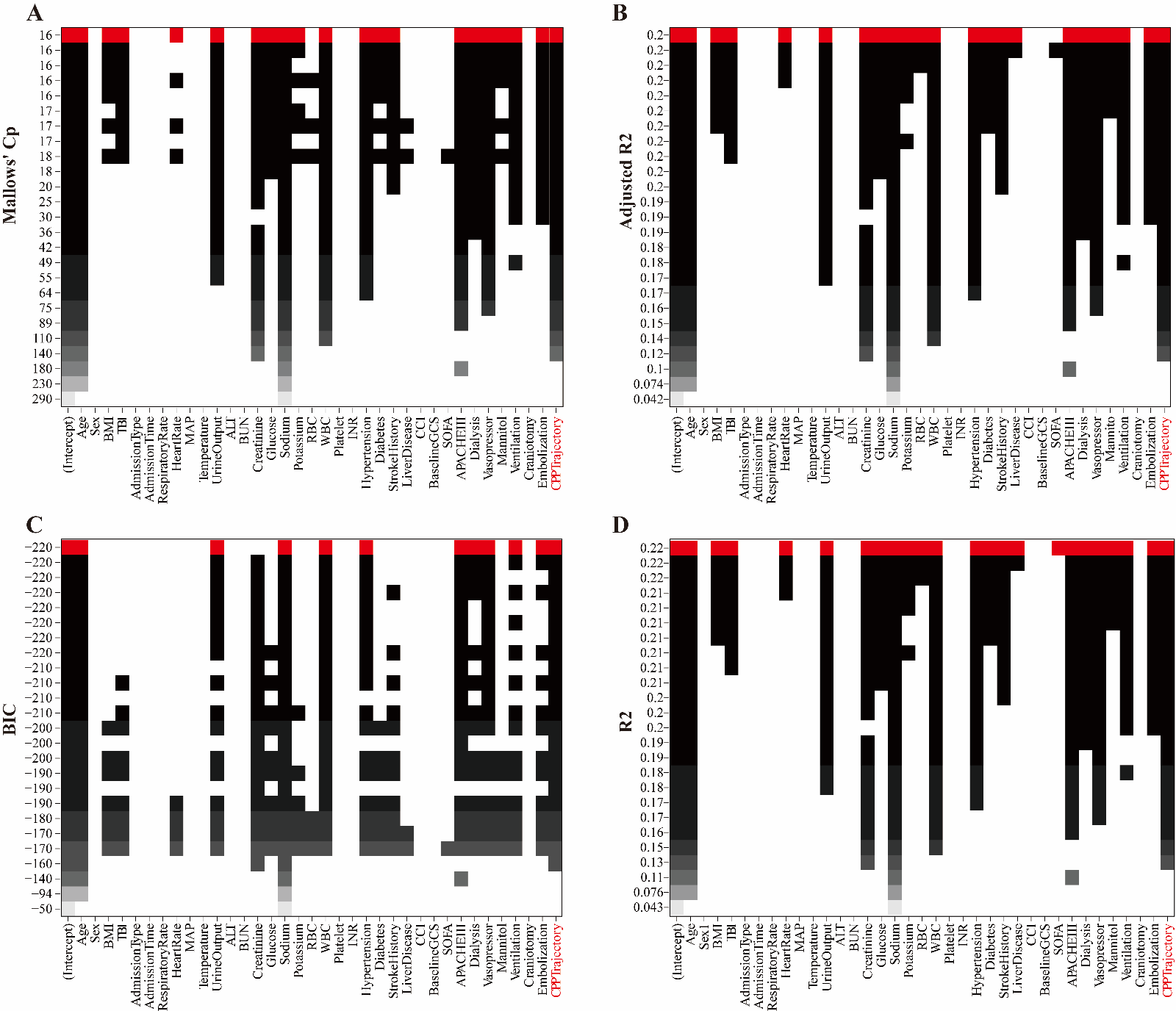


**Legend:** Variable selection patterns under best subset selection (BSS) criteria: (A) Mallows’ Cp, (B) adjusted R², (C) BIC, and (D) R². The top row highlighted in red indicates the optimal subset under each criterion, whereas black and gray denote less optimal subsets. CPP trajectory phenotype was retained in the optimal subset across all four BSS criteria.

# Supplementary Figure 15. Consensus and Co-selection Patterns of Candidate Predictors Across Feature Selection Methods


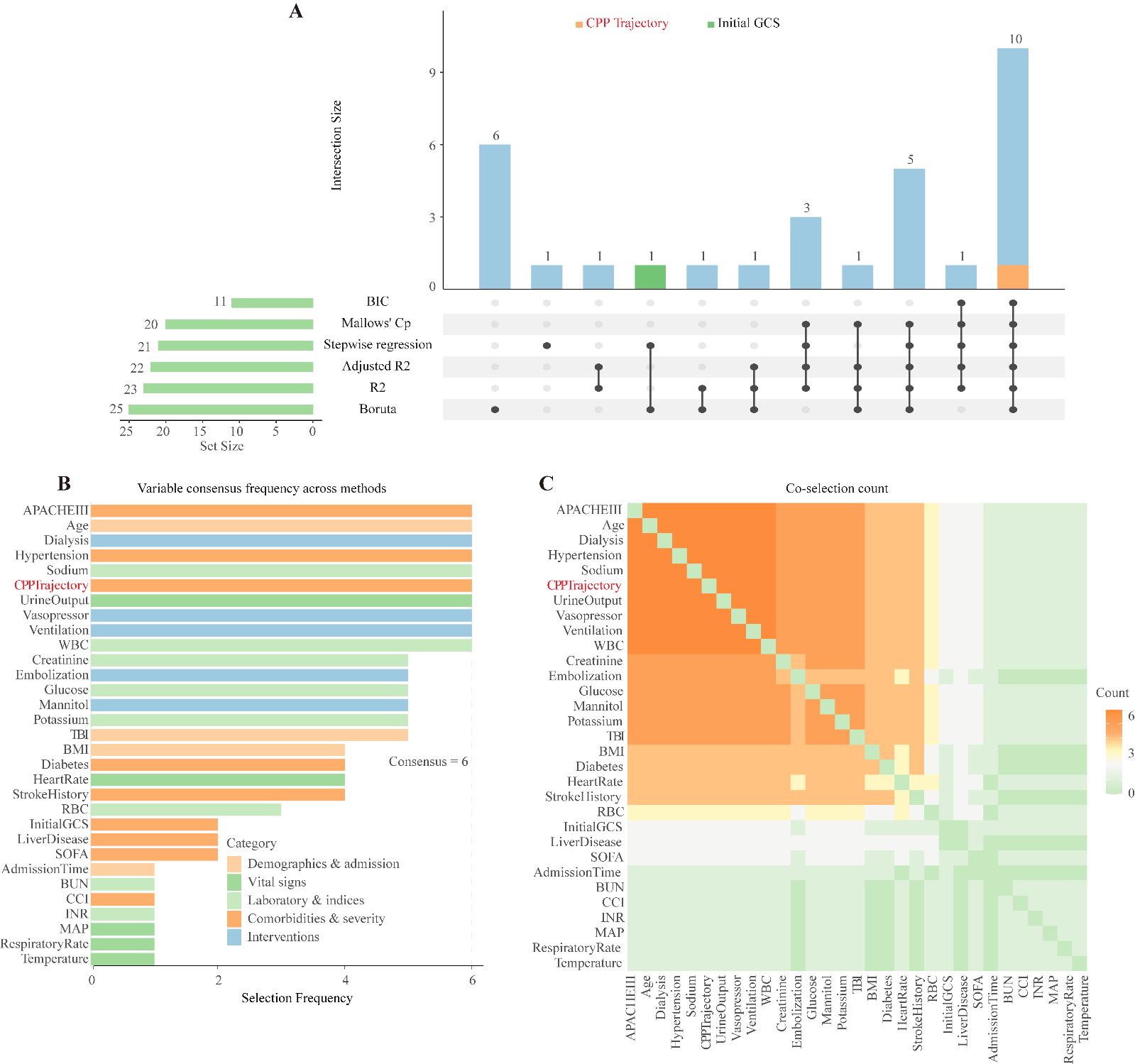


**Legend:** Panel A shows an UpSet plot of overlaps among variables selected by Boruta, stepwise regression, and best subset selection under the BIC, Mallows’ Cp, adjusted R², and R² criteria; vertical bars indicate intersection sizes, and horizontal bars indicate the total number of variables selected by each method. Panel B shows the selection frequency of each candidate predictor across methods, with bar colors indicating variable categories. Panel C shows pairwise co-selection counts among candidate predictors, where warmer colors indicate more frequent co-selection. CPP trajectory showed high consensus across methods and frequent co-selection with other key predictors.

# Supplementary Figure 16. Performance of the Trajectory-Enhanced Model for In-Hospital Mortality in the Training Cohort

**
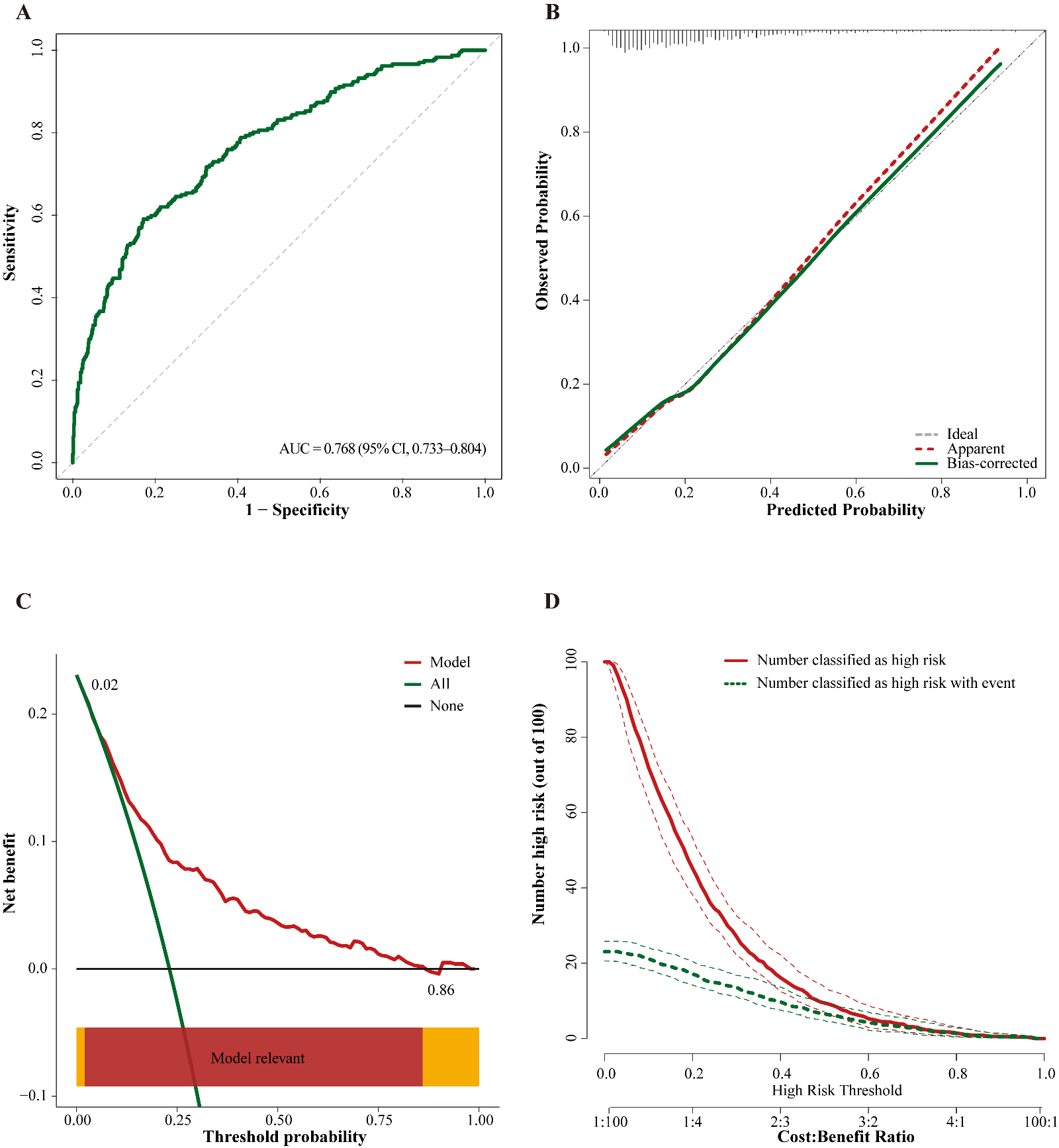
**

**Legend:** The trajectory-enhanced model included age, urine output, sodium, WBC, hypertension, APACHE III score, dialysis, vasopressor use, mechanical ventilation, and CPP trajectory phenotype. Panel A shows the ROC curve with the area under the curve (AUC) and 95% confidence interval. Panel B shows the bootstrap-corrected calibration curve based on 1,000 bootstrap resamples, with the ideal, apparent, and bias-corrected lines. Panel C shows decision curve analysis of net benefit across threshold probabilities. Panel D shows the clinical impact curve, including the number classified as high risk and the number classified as high risk with events across threshold probabilities.

# Supplementary Figure 17. Predictive Performance of the Trajectory-Enhanced Model in the Test Cohort

**
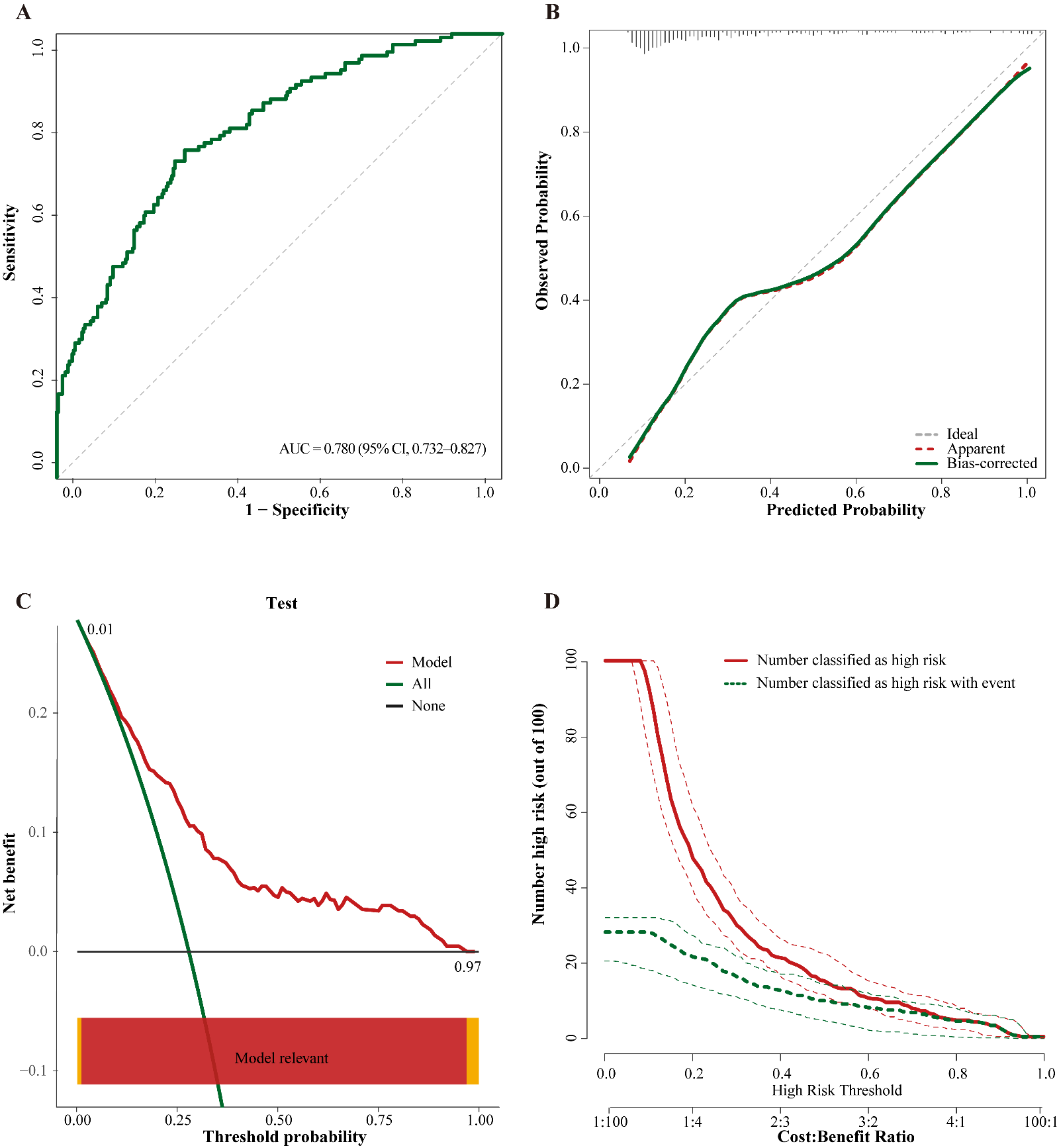
**

**Legend:** Panel A shows the ROC curve with the AUC and 95% CI. Panel B shows the bootstrap-corrected calibration curve with the ideal, apparent, and bias-corrected lines. Panel C shows DCA across threshold probabilities. Panel D shows the clinical impact curve, including the number classified as high risk and the number classified as high risk with events.
